# Interplay of Seasonality, Major and Trace Elements: Impacts on the Polychaete *Diopatra neapolitana*

**DOI:** 10.3390/biology11081153

**Published:** 2022-07-31

**Authors:** Valéria Giménez, Paulo Cardoso, Carina Sá, Carla Patinha, Eduardo Ferreira da Silva, Etelvina Figueira, Adília Pires

**Affiliations:** 1 Department of Biology, University of Aveiro, 3810-193 Aveiro, Portugal; valeriacgimenez@ua.pt; 2 Centre for Environmental and Marine Studies (CESAM), Department of Biology, University of Aveiro, 3810-193 Aveiro, Portugal; pjcardoso@ua.pt (P.C.); carinafsa@ua.pt (C.S.); efigueira@ua.pt (E.F.); 3 GEOBIOTEC, Department of Geosciences, University of Aveiro, 3810-193 Aveiro, Portugal; cpatinha@ua.pt (C.P.); eafsilva@ua.pt (E.F.d.S.)

**Keywords:** *Diopatra neapolitana*, biochemical endpoints, oxidative stress, major elements, trace elements, seasonality

## Abstract

**Simple Summary:**

Coastal systems often serve as sinks for toxic elements, and seasonality has been responsible for many changes in the physical and chemical parameters of waters and sediments, leading to geochemical alterations in aquatic systems and the alteration of element uptake rates in organisms. *Diopatra neapolitana* worms were collected from five sites of the Ria de Aveiro lagoon in the autumn, winter, spring, and summer of 2018/2019 and were tested to check for differences in the biochemical responses (cell damage, antioxidant enzymes, biotransformation enzymes, and energy-related parameters) among seasons and sites. In general, the results demonstrated that enzyme activities were higher in spring and summer due to high temperatures and element bioaccumulation. Energy-related parameters presented with higher levels in spring and autumn, which was mainly due to element bioaccumulation. Oxidative damage was higher during winter and was related to salinity and decreases in temperature. This study demonstrated that abiotic factors influence the geochemistry of elements and that both significantly affect organism performance in low-contamination systems, such as the Ria de Aveiro lagoon. This knowledge is important to understand how ecological and economically relevant species such as *D. neapolitana* respond to environmental changes.

**Abstract:**

Polychaetes are known to be good bioindicators of marine pollution, such as inorganic contamination. Major and trace elements are commonly present in sediment and may be accumulated by polychaetes such as the tubiculous *Diopatra neapolitana*. In this study, *D. neapolitana* individuals were collected in the autumn, winter, spring, and summer of 2018/2019 from the Ria de Aveiro lagoon (western Portugal) to understand how seasonality influences element accumulation. The impact of the interaction of seasonality and elements on oxidative status, energy metabolism, and oxidative damage was also assessed. The obtained results showed that the activity of the antioxidant enzymes catalase, glutathione S-transferases, and non-protein thiol levels were higher in summer and that superoxide dismutase, lipid peroxidation, and electron transport system activity increased in winter. The lowest glycogen levels were observed during spring, and protein carbonylation was the highest during autumn. These results could mainly be related to high temperatures and the bioaccumulation of Al, As, Mn, and Zn. Energy-related parameters increased during spring and autumn, mainly due to the bioaccumulation of the same elements during spring and summer. Lipid damage was higher during winter, which was mainly due to salinity and temperature decreases. Overall, this study demonstrates that seasonality plays a role in element accumulation by polychaetes and that both impact the oxidative status of *D. neapolitana.*

## 1. Introduction

Coastal zones usually serve as sinks for toxic elements due to the discharge of effluents resulting from anthropogenic activity. Ria de Aveiro, a shallow estuarine ecosystem located on the northeast coast of Portugal (40°38′ N, 8°45′ W), constitutes a permanent habitat for various species of fauna and flora. There are many activities in Ria de Aveiro, ranging from economical (e.g., salt production, aquaculture, fisheries, agriculture) to touristic activities [1]. Ria de Aveiro is a good example of an ecologically active coastal system that has been suffering more and more over the years from anthropogenic activities such as agriculture and the discharge of effluents containing various toxic elements (e.g., elements, polycyclic aromatic hydrocarbons, and pharmaceuticals), which have contributed to the degradation of the ecosystem and water quality [2,3,4,5].

Trace metals and metalloids exist naturally in the environment, but in certain amounts or with alterations in their configuration due to physical and chemical changes in water, they can become toxic and have deleterious effects on marine organisms (e.g., [6,7,8,9,10,11]). Although metals have a natural origin, they can also have anthropogenic origins, such as mining and smelting operations, refining and electroplating, dye and paint manufacture, and fossil fuel burning [12]. Organisms that are both in contact with sediment and water, such as polychaetes, are more conducive to accumulating these elements and suffering from the physical and chemical changes that occur in their environment [13]. Increased reactive oxygen species, the inhibition of antioxidant enzymes, and decreased fecundity and mortality are some of the various impacts that metals have on these organisms [14,15,16]. Laboratorial exposure of *Perinereis aibuhitensis* to Cd suggested that this element interfered with the antioxidant defence system of the studied polychaete [17]. Bouraoui et al. [18] also observed the induction of oxidative stress biomarkers in different body regions of the polychaete *Hediste diversicolor* when exposed to Cu. A field study carried out along Ria de Aveiro demonstrated that various metals (chromium (Cr), nickel (Ni), copper (Cu), lead (Pb), cadmium (Cd), mercury (Hg)), and Arsenic (As)) accumulated in *D. neapolitana,* leading to cellular damage and increased antioxidant and to the biotransformation of enzyme activity [13]. The same study revealed that the elements that were analysed predominantly bioaccumulated in the insoluble fraction present in the precipitated form or in the fraction that is bound to membranes, thus not causing toxicity to organisms [19]. Pires et al., [13] also stated that Cu, Cr, Pb, Ni, and As were the most abundant elements (26.22, 16.95, 8.61, 5.33, 4.48 mg/kg DW, respectively), while Cd and Hg were the least abundant (0.02 and 0.01 mg/kg DW, respectively).

Invertebrates such as polychaetes are widely used in impact assessment studies for scenarios with stress conditions caused by pollutants or climate change since they play an important role in the food chain [20,21]. Therefore, this group of organisms can help us to infer how damaged the environment in which they live could be. Polychaetes are usually the most abundant taxon in benthic communities [22,23] and have a wide geographical range [24,25,26]. Their life cycle is not very complex, they are easy to collect and maintain in laboratory conditions, and they usually live buried in sediment, making them highly exposed to contaminants [27,28]. The tubiculous onuphid polychaete *Diopatra neapolitana* presents a wide geographical distribution and has been reported in several intertidal and shallow subtidal habitats [26,29,30] and is widespread in the Ria de Aveiro lagoon, being present in all of the principal channels [31]. The development of *D. neapolitana* includes a larval phase (lecithotrophic free-swimming larvae), with the main reproduction period spanning from June to August [32]. Gametes develop in the coelom cavity and after fecundation occurs in the water column, which is where fecundation occurs [32]. This species plays an important ecological role since it constitutes an important food source for birds, fish, and crustacea populations. Additionally, their tubes contribute to sediment stabilization and generate eddies that enhance surface scour and transport large organic particles, increasing the sediment’s structural complexity and its biodiversity by providing refuge from disturbance and predation [33]. Additionally, the worm and its tube facilitate the settlement of some algae species [34], and furthermore, this species is economically important, as it is intensively collected by bait diggers to be used in fisheries as fish bait [26]. This species inhabits a tube built by the organism, and it is buried in sediment most of the time [34]. *D. neapolitana* has been shown to be a good bioindicator of inorganic contamination [13], organic enrichment [35], pharmaceutical [36] and water acidification, temperature shifts, and salinity fluctuations [37,38,39].

Previous studies also indicated that environmental fluctuations, such as temperature and salinity fluctuations, impact polychaetes, and impacts such as changes in survivorship, regenerative capacity, oxidative stress, and energy reserves have been documented [37,38,39]. Thus, in ecosystems with low levels of pollution, it can be difficult to determine if the observed effects are due to contaminants or to natural changes that are closely linked to the life cycle of the organism [13,40]. Since organism responses to stressful conditions can be measured at the subcellular level through biochemical analysis [10,41,42,43,44,45], this study aims to evaluate the impact of element contamination in *D. neapolitana* species harvested from different sites of the Ria de Aveiro lagoon during the four seasons of the year. We tested whether there is a seasonal effect on the impact of contaminants on the performance of the polychaete *D. neapolitana* by collecting samples from five different locations (Cale do Ouro, Murtosa, São Jacinto, Torreira, and Costa Nova) over the four seasons of the year. The biochemical performance of *D. neapolitana* organisms (membrane-related parameters, antioxidant defences, and oxidative damage) was assessed. We expected to detect alterations in these parameters, considering that previous studies demonstrated that trace element accumulation, salinity changes, and temperature increases induced antioxidant defence activity and caused membrane and protein damage in polychaetes when considered alone [38,39,45]. Thus, it is critical to understand the interaction between seasonality and a species’ response to contaminants in order to interpret the experimental results.

## 2. Materials and Methods

### 2.1. Study Area, Sampling and Measurement of Physic-Chemical Parameters

During the autumn of 2018 and the winter, spring, and summer of 2019, polychaetes were collected from five different sites (Cale Do Ouro—CD, Murtosa—M, São Jacinto—SJ, Torreira—T, and Costa Nova—CN) of the Ria de Aveiro lagoon (western Portugal) (Figure S1). The Ria of Aveiro is a shallow coastal lagoon located on the Atlantic coast of northwest Portugal (40°38′ N, 8°44′ W) and is separated from the sea by a sand bar and is characterized by narrow channels and the existence of significant intertidal areas, namely mudflats and salt marshes [46]. The collected polychaetes were transported to the laboratory in ice-cold plastic containers and were frozen at −20 °C until further analysis (biochemical endpoints and elements bioaccumulation).

### 2.2. Samples Analysis

#### 2.2.1. Environmental Parameters

In situ measurements of the pH, redox potential (Eh), dissolved oxygen (DO), salinity, and temperature were performed at the sediment–water interface using a handheld multiparametric waterproof meter (HI98194—Hanna Instruments) in each sampling area and season (Table S1). Sediments were also collected from each area and transported to the laboratory and used for grain-size analysis (% fine particles), organic matter content determination (% OM), and major and trace element quantification.

Sediment grain size was analysed in the sites from which *D. neapolitana* was collected by wet and dry sieving following the procedure outlined in Quintino et al. (1989) [47]. The sediment was classified according to the median value (P50) according to the Wentworth scale [48]: very fine sand (median between 0.063 and 0.125 mm); fine sand (0.125–0.250 mm); medium sand (0.250–0.500 mm), or coarse sand (0.500–1 mm). The final sediment classification adopted the descriptors “clean“, “silty”, or “very silty” when fine particles (particles with a diameter below 0.063 mm) were below 5%, from 5 to 25%, and from 25 to 50% of the total sediment dry weight, respectively [47].

The total organic matter content was determined by weight loss on ignition at 450 °C over 5 h [49] for a 1 g sediment sample after an initial drying session at 60 °C for 24 h. The percentage of fine particles was determined by wet sieving through a 0.063 mm mesh screen following the method used in Quintino et al. (1989) [47].

#### 2.2.2. Quantification of Major and Trace Elements in Sediments and Tissue Samples

The concentrations of aluminium (Al), arsenic (As), barium (Ba), beryllium (Be), calcium (Ca), cadmium (Cd), cobalt (Co), chromium (Cr), copper (Cu), iron (Fe), potassium (K), lithium (Li), magnesium (Mg), manganese (Mn), sodium (Na), nickel (Ni), phosphorus (P), lead (Pb), antimony (Sb), tin (Sn), thallium (Tl), vanadium (V), tungsten (W), and zinc (Zn) were analysed in sediments and organisms (soluble and insoluble fractions) by inductively coupled plasma mass spectrometry (ICP-MS) (Agilent 7700x, Santa Clara, CA, USA) after acid digestion. All of the elements were analysed and quantified using the equipment.

Regarding the sediments, a 250 mg sample of dried and homogenized sediment was mixed with 1.5 mL of HCl (37%) (Fisher Chemical) and 4.5 mL of HNO3 (65%) (Fisher Chemical, Hampton, NH, USA) in DigiPrep tubes. After reacting with acid overnight (14–16 h), the solutions were heated on a digestion block (DigiPrep, SCP Science, Montreal, QC, Canada). These solutions were initially exposed for 10 min and then exposed to an increase from room temperature to 50 °C that was kept stable for 15 min. The temperature was then increased from 50 °C to 85 °C for a period of 15 min. When the temperature stabilized at 85 °C, it was maintained for 15 min until the end of the cycle. After the digestion process, 45× mL of ultrapure water was added, and the tubes were centrifuged for 20 min at 3500 g (Heraeus Multifuge 3 S-R, Hanau, Germany) and then analysed by ICP-MS. A rigorous quality control procedure was performed during these analyses, which included the analysis of the blanks, duplicate samples, and certified reference materials. The accuracy of the ICP-MS and digestion method was evaluated via the analysis of the certified reference materials ERM-CC141 LOAM SOIL and Till-2. The precision and bias error of the chemical analysis was less than 10%.

Regarding the tissue samples, 500 mg of homogenized tissue was subjected to subcellular fractionation by centrifugation at 1450× g for 15 min at 4 °C. Fractionation resulted in the isolation of two distinct fractions: insoluble and soluble. The insoluble fraction can be defined as the element concentration that is unavailable and that is precipitated in insoluble metal-rich granules and in cellular debris, while the soluble fraction can be defined as the element concentration in its free form or that is bound to the proteins present in the cytosol [50]. Each fraction was dried at 40 °C, and then 1.5 mL of HCl (37%) and 4.5 mL of HNO3 (65%) were added to each fraction in Teflon vessels. After 24 h, the Teflon vessels were placed on a heating plate at 115 °C, and after 6 h, 45 mL of ultrapure water was added, and the contents were transferred to falcon tubes that were centrifuged for 20 min at 3500 g and then analysed by ICP-MS. Rigorous quality control was performed during these analyses, which included the analysis of the blanks, duplicate samples, and certified reference materials. The precision and bias error of the chemical analysis was less than 10%.

The concentrations of the elements were expressed in mg kg^−1^ DW since the sediments and tissue fractions were previously dried over 48 h at 40 °C. To assess the ability of the polychaetes to accumulate elements, the biota–sediment accumulation factor (BSAF) was determined according to [51]. BSAF = Ct/Cs, where Ct is the element concentration in the polychaete tissues, and Cs is the element concentration in the sediment.

#### 2.2.3. Biochemical Parameters

Frozen specimens (9 organisms per area, per season) were homogenized with a 170 mortar and a pestle in liquid nitrogen and were divided into subsamples. Extraction was performed in the proportion of 1:2 *w*/*v* in phosphate buffer containing 50 mM of potassium phosphate (pH = 7), 1 mM of ethylenediamine tetra acetic acid (EDTA), 1% (*v*/*v*) Triton X-100, and 1 mM of dithiothreitol (DTT) for the protein (PROT), glycogen (GLY), catalase (CAT), superoxide dismutase (SOD), glutathione S-transferases (GST), non-protein thiol (NPT), and protein carbonylation (ProC) assays. For electron transport system (ETS) determination, sample extraction was carried out in 0.1 M of Tris–HCl pH 8.5, 15% (*w*/*v*) polyvinylpyrrolidone (PVP), 153 μM of magnesium sulphate (MgSO4), and 0.2% (*v*/*v*) Triton X-100. Extraction with 20% (*v*/*v*) trichloroacetic acid (TCA) was performed for lipid peroxidation (LPO) determination.

Samples were centrifuged for 20 min at 3000× g at 4 °C for the electron transport system, and the remaining samples were centrifuged for 20 min at 10,000× g at 4 °C. The samples used for glycogen (GLY) and non-protein thiol (NPT) determination were not centrifuged. Supernatants were stored at −20 °C or were used immediately. All of the biochemical parameters were determined in duplicate. 

#### 2.2.4. Metabolism-Related Parameters

Protein (PROT) was measured following the Biuret method [52] using bovine serum albumin (BSA) (Sigma-Aldrich, Saint Louis, MO, USA) as a standard (0–40 mg mL^−1^). The absorbance was recorded at 540 nm (Tecan Infinite 200 Pro microplate reader), and the total protein content was expressed in mg g^−1^ of fresh weight (FW).

Glycogen (GLY) quantification was carried out according to [53] using phenol (Sigma-Aldrich, Saint Louis, MO, USA) and sulfuric acid (LabChem, Zelienople, PA, USA). The absorbance was recorded at 492 nm, and the results were expressed in mg g^−1^ of FW. 

The electron transport system (ETS) was measured following the method described by [54] with modifications [55]. Absorbance was recorded at 490 nm for 10 min with 25 s readings. The amount of formazan formed was calculated using ɛ = 15.900 M^−1^ cm^−1^, and the results were expressed in nmol min^−1^ per g FW. 

#### 2.2.5. Antioxidant Defences

Catalase (CAT) activity was quantified according to [56]. Formaldehyde (Sigma-Aldrich, Saint Louis, MO, USA) standards (0–150 μM) were used for the standard curve. Absorbance was measured at 540 nm. The enzymatic activity was expressed in U g^−1^ FW (U = 1 nmol min^−1^).

The activity of the enzyme superoxide dismutase (SOD) was determined based on the method in [57]. SOD (Sigma-Aldrich, Saint Louis, MO, USA) standards (0–60 U mL^−1^) were used for the standard curve. Following incubation, SOD activity was measured spectrophotometrically at 560 nm. The activity of this enzyme was expressed in Ug FW.

The activity of the glutathione S-transferase (GST) isoenzymes was determined according to [58]. This reaction was measured by following the increasing absorbance at 340 nm and by taking readings every 15 s over 5 min. GST activity was expressed in U g^−1^ FW using ε = 9.6 mM^−1^ cm^−1^.

Non-protein thiols (NPTs) were measured according to the method described by [59] and adapted by [60] and described by [61]. Absorbance was read at 412 nm, and the results were expressed in nmol g^−1^ of FW.

#### 2.2.6. Oxidative Damage Endpoints

Lipid peroxidation (LPO) was measured according to [62]. Absorbance was read at 532 nm, and the analysis was carried out by calculating the malondialdehyde (MDA) concentration using ε = 1.56 × 105 M^−1^ cm^−1^. LPO levels were expressed in nmol of MDA formed per g of fresh weight.

The protein carbonylation (ProC) parameter was measured following the methods described in [63] and [64]. The absorbance was read at 450 nm, and the protein-conjugated hydrazine was calculated using ε = 22.308 mM^−1^ cm^−1^. Results were expressed in g^−1^ of FW. 

### 2.3. Statistical Data Analysis

Biochemical descriptors (PROT content, GLY, ETS, CAT, SOD, GSTs, NPT, LPO, and ProC) were submitted to hypothesis testing using permutational multivariate analysis of variance by employing the PERMANOVA+ add-on in PRIMER v6 (Anderson et al. 2008). For each descriptor, significant differences were assessed for all areas and seasons. All of the descriptors were analysed following a one-way hierarchical design, with the areas as the main fixed factor. The null hypotheses that were tested were (a) for each element, no significant differences exist among areas and seasons; (b) for each biochemical parameter (PROT, GLY, ETS, CAT, SOD, GSTs, NPT, LPO, and ProC), no significant differences exist among areas and seasons. Significance levels (*p* ≤ 0.05) among sampling areas and among seasons were presented with different letters.

Data from the biochemical and environmental parameters (pH, salinity, temperature, DO, Eh, and % of fine particles and % OM per area and per season) were transformed (square root), normalized, and used to calculate a Euclidean matrix between sampling areas. Principal coordinates ordination analysis (PCO) was used to visualize the differences among areas. The environmental parameters that were highly correlated (r > 0.7) were represented as superimposed vectors in the graph.

Afterwards, the matrix containing data from the biochemical parameters and physical–chemical data of the sampling areas, the bioaccumulation of elements in their soluble fraction (elements present in the cytosol and that are available for organisms) in *D. neapolitana* per sampling area, and the season was used to perform another PCO analysis. The variables presenting a correlation higher than 70% with sample ordination were represented as superimposed vectors in the PCO graph.

Data obtained from the biota–sediment accumulation factor (BSAF) regarding element accumulation in sediments and tissues were transformed (logarithmic transformation) using the program Metaboanalyst. These data were represented in heatmaps.

## 3. Results

### 3.1. Physical and Chemical Characteristics of Sediment Samples

Overall, summer had higher salinity levels at all sites, and SJ, T, and CN had the lowest levels during autumn (Table S1). The lowest DO level, 6.03 mg/L, was found at site M during spring, and the highest, 9.75 mg/L, was found at site CN in spring. Eh varied between 67.27 mV and 255.57 mV, with SJ presenting the lowest values during summer and site T presenting the highest values during spring. The lowest temperature levels were recorded during winter, and pH levels did not vary significantly between seasons and sites. The highest values of % OM, 6.30, were observed during autumn in CD and in summer in CN. The lowest values were recorded during spring and summer in SJ. Regarding the % of fine particles, these values varied significantly among sites and seasons, with the highest value being recorded in autumn in CD and CN in summer, and the lowest values being recorded in CN in autumn (Table S1). No values are available for site M during winter because it was not possible to collect samples due to the climacteric conditions. Site CD was classified as muddy in autumn and as comprising silty very fine sand in the remaining seasons; site M was classified as having very silty fine sand in autumn and silty fine sand in spring and summer; CN was classified as having clean fine sand in autumn and silty fine sand in the remaining seasons; T was classified as having silty medium sand in autumn and summer and as having very silty fine sand in winter and spring. SJ was classified as having very silty fine sand in autumn and winter and silty fine sand in spring and summer.

**Figure 1:**
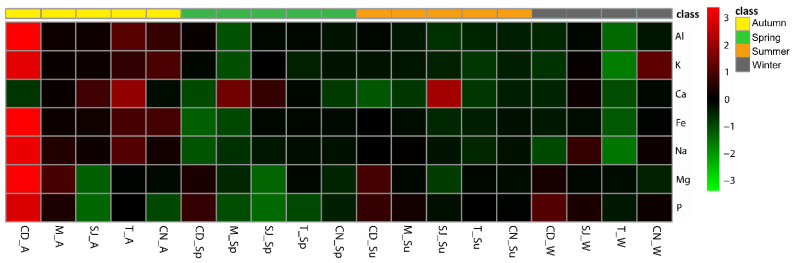
Heatmap comparing major element concentrations (Al, Ca, Fe, K, Mg, Na, and P) in sediments from sampling sites. CD, M, SJ, T, and CN represent the sites Cale do Ouro, Murtosa, São Jacinto, Torreira, and Costa Nova, respectively. A, W, Sp, and Su represent the seasons of the year: autumn, winter, spring, and summer.

**Figure 2:**
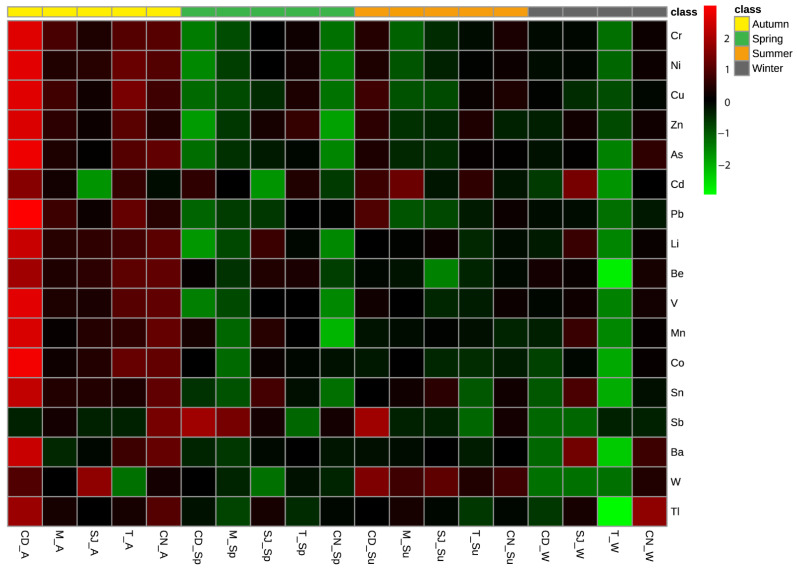
Heatmap comparing trace element concentrations (As, Ba, Be, Cd, Co, Cr, Cu, Li, Mn, Ni, Pb, Sb, Sn, Tl, V, W, and Zn) in sediments from sampling sites. CD, M, SJ, T, and CN represent the sites Cale do Ouro, Murtosa, São Jacinto, Torreira, and Costa Nova, respectively. A, W, Sp, and Su represent the seasons of the year: autumn, winter, spring, and summer.

In autumn, site CD showed the highest concentration of all of the major elements, with the exception of Ca, in its sediments (Figure 1). Overall, in autumn, a higher concentration of major elements was observed for all of the sites, and in spring, a lower concentration of these elements was observed. Regarding the trace element concentration in the sediments, generally, the concentrations were higher in autumn and lower in winter and spring. Site T in winter had the lowest concentration of trace elements, and site CD in autumn had the highest concentration (Figure 2). 

### 3.2. Elements Concentration in Sediments

In autumn, site CD showed a higher concentration of all of the major elements with the exception of Ca, in its sediments. Overall, a higher concentration of major elements was observed for all of the sites in autumn, and in spring, a lower concentration of these elements was observed (Figure 1). 

Regarding the trace element concentrations in the sediments, generally, the concentrations were higher in autumn and lower in winter and spring. Site T in winter had the lowest concentration of trace elements, and site CD in autumn had the highest concentration (Figure 2). The most contaminated site was site CD.

### 3.3. Bioaccumulation of Elements in Polychaetes

**Figure 3:**
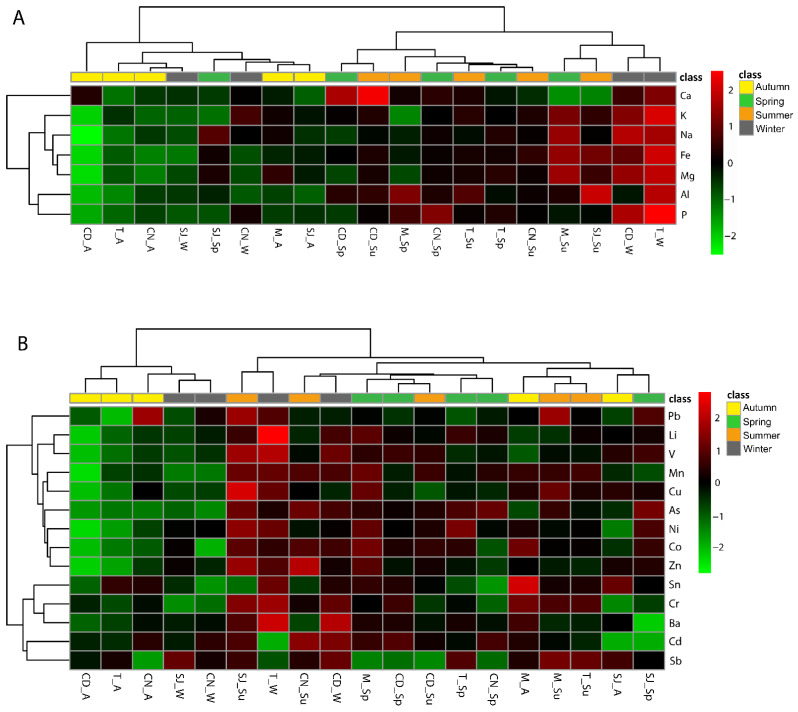
Heatmap with the biota–sediment accumulation factor (BSAF): (**A**) major BSAF (Al, Ca, Fe, K, Mg, Na, and P) and (**B**) trace element BSAF (As, Ba, Cd, Co, Cr, Cu, Li, Mn, Ni, Pb, Sb, Sn, V, and Zn) in *Diopatra neapolitana* tissues and sediments of the sampling sites. CD, M, SJ, T, and CN represent the sites Cale do Ouro, Murtosa, São Jacinto, Torreira, and Costa Nova, respectively. A, W, Sp, and Su represent the seasons of the year: autumn, winter, spring, and summer. The clusters in the figures allow the most similar responses to be grouped together.

Regarding the bioaccumulation of major elements in the tissue samples of the polychaetes, there was a clear separation between the concentrations of these elements in the soluble and insoluble fractions (Figure S2). Major elements such as Al, Ca, Fe, Mg, and P were higher in the insoluble fraction, while Na and K were found in higher concentrations in the tissues of the soluble fraction. There were no significant differences in major elements bioaccumulation between seasons. In autumn, all of the sites had generally low values of the biota–sediment accumulation factor (BSAF), and the Cale do Ouro, Torreira, and Murtosa were the sites with the lowest BSAF values. On the other hand, the sites Cale do Ouro and Torreira had higher values in winter. The other seasons and sites had intermediate BSAF values (Figure 3A).

Regarding the bioaccumulation of trace elements in the polychaete samples, it was possible to observe higher bioaccumulation in the insoluble fraction of the tissues regardless of the season and site compared to the soluble fraction. However, site SJ in spring and summer had high concentrations of trace metals in both the soluble and insoluble fractions (Figure S3). The element value of Be was under the detection limit and is therefore not represented in the heatmap. Overall, Zn was the element that organisms accumulated the most. The site where the organisms accumulated the most was SJ, and the seasons in which that accumulation occurred were mainly autumn and summer.

Regarding the BSAF concerning trace elements, the sites with the lowest values were Cale do Ouro and Torreira. The highest values were mostly recorded in winter in Cale do Ouro, São Jacinto, and Torreira (Figure 3B). 

### 3.4. Metabolism-Related Parameters

**Figure 4:**
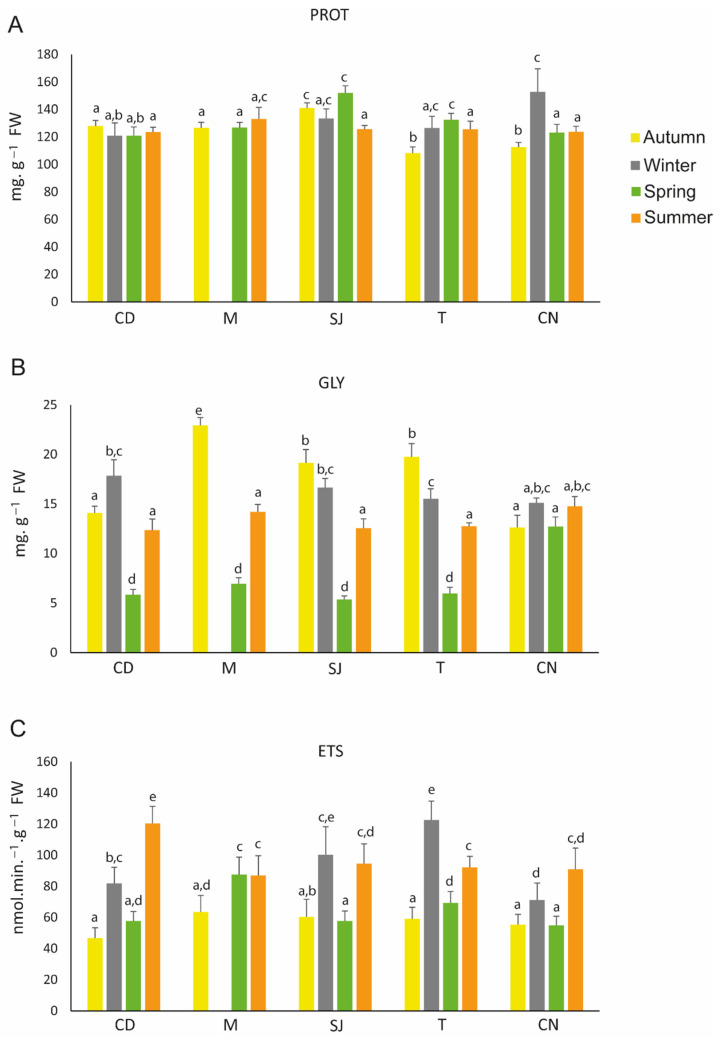
Metabolism-related parameters: (**A**) protein (PROT), (**B**) glycogen (GLY), and (**C**) electron transport system (ETS) measured in *Diopatra neapolitana* in autumn 2018 and winter, spring, and summer 2019. Comparison between different seasons at the same site. Letters (a–e) represent significant differences between areas and seasons (*p* ≤ 0.05). CD: Cale do Ouro; M: Murtosa; SJ: São Jacinto; T: Torreira; CN: Costa Nova. Site M has no winter data.

The protein (PROT) content was lower in organisms from sites T and CN in autumn and was higher in organisms from site SJ in spring and site CN in winter (Figure 4A; Table S2). Regarding the glycogen (GLY) concentrations, in almost all areas, significant differences were observed among seasons, with site CN presenting few differences (Figure 4B; Table S2). In general, electron transport system (ETS) activity decreased in organisms collected in autumn and in spring for all sites, except in site M and in winter in CN, where no significant differences were observed (Figure 4C; Table S2).

### 3.5. Antioxidant Defences

**Figure 5:**
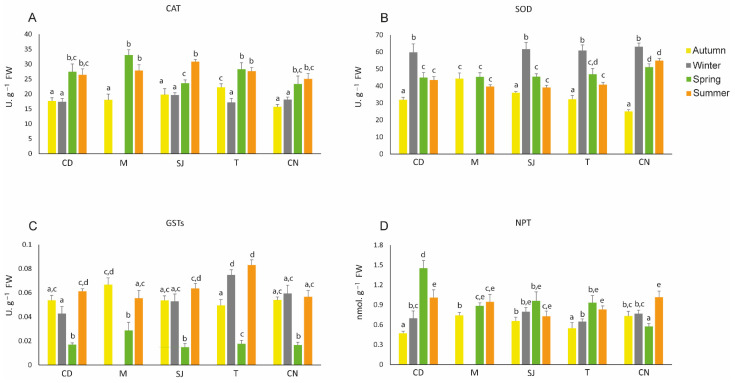
Antioxidant defences: (**A**) catalase (CAT), (**B**) superoxide dismutase (SOD), (**C**) glutathione S-transferases, and (**D**) non-protein thiols (NPTs) measured in *Diopatra neapolitana* in autumn 2018 and winter, spring, and summer 2019. Comparison between different seasons at the same site. Letters (a–e) represent significant differences between areas and seasons (*p* ≤ 0.05). CD: Cale do Ouro; M: Murtosa; SJ: São Jacinto; T: Torreira; CN: Costa Nova. Site M has no winter data.

Catalase (CAT) activity generally increased in spring and summer in all areas. This increase was significant for all sites and seasons, except in SJ in autumn and spring and in CN in winter and spring (Figure 5A; Table S2). Superoxide dismutase (SOD) activity generally increased in all sites during winter and was lower in organisms from CN during autumn. The lower activity during autumn in CN was also significantly different (Figure 5B; Table S2). GST activity was significantly lower in all sites during spring and significantly higher during winter and summer in site T (Figure 5C; Table S2). Generally, non-protein thiols (NPTs) were lower during autumn, except in site CN, and higher during spring, except in sites M and CN. The lowest value was obtained during autumn in CD (Figure 5D; Table S2).

### 3.6. Oxidative Damage Endpoints

**Figure 6:**
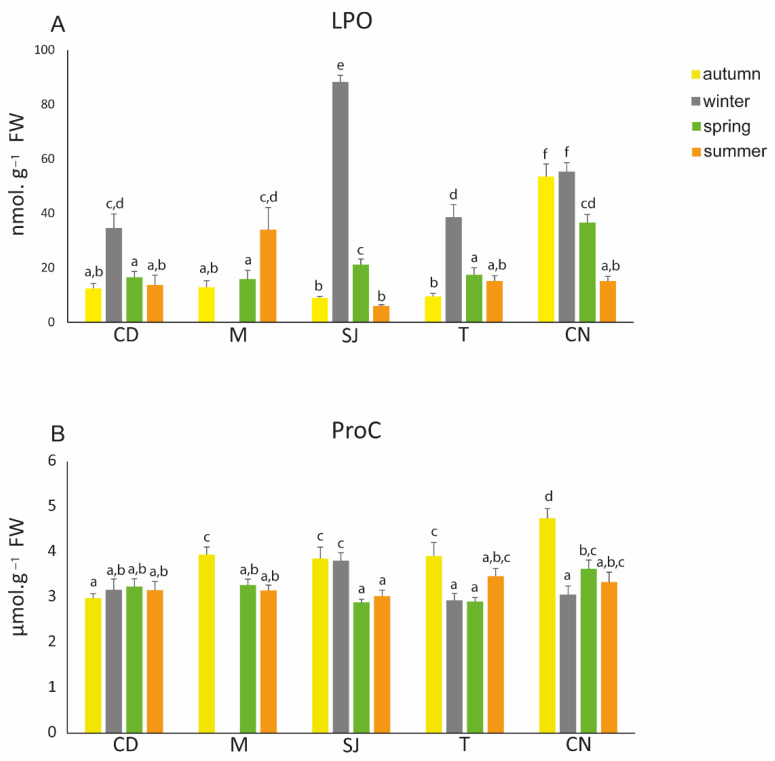
Oxidative damage endpoints: (**A**) lipid peroxidation (LPO) and (**B**) protein carbonylation measured in *Diopatra neapolitana* in autumn 2018 and winter, spring, and summer 2019. Comparison between different seasons. Letters (a–f) represent significant differences between areas and seasons (*p* ≤ 0.05). CD: Cale do Ouro; M: Murtosa; SJ: São Jacinto; T: Torreira; CN: Costa Nova. Site M has no winter data.

Lipid peroxidation (LPO) levels were significantly higher during winter, especially in SJ, where all of the LPO levels were significantly different among seasons. Additionally, the lowest levels of LPO were seen in summer in SJ. Generally, LPO levels were low during autumn, except in CN, where they were significantly higher (Figure 6A; Table S2). ProC was higher in all sites during autumn, except in CD, where no significant differences between seasons were observed. Additionally, in autumn, levels were significantly higher in CN. During winter in SJ, levels were higher compared to the other sites (Figure 6B; Table S2).

### 3.7. Multivariate Analysis as a Tool to Summarise Information

#### 3.7.1. Elements and Physical–Chemical Data

**Figure 7:**
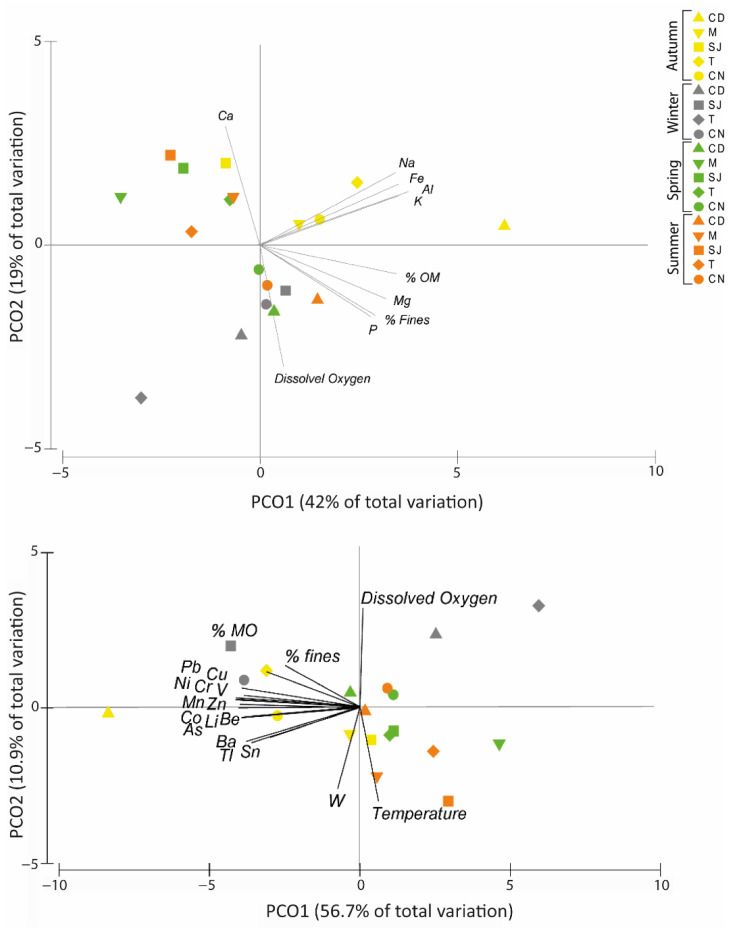
Principal coordinates ordination analysis (PCO) of (**A**) major element concentrations in sediments and physical and chemical parameters and (**B**) trace element concentrations in sediments and physical and chemical parameters. (r > 0.7). CD, M, SJ, T, and CN represent the sites Cale do Ouro, Murtosa, São Jacinto, Torreira, and Costa Nova, respectively. A, W, Sp, and Su represent the seasons of the year: autumn, winter, spring, and summer.

Principal coordinates ordination analysis (PCO) of the major element concentrations in the sediments and the physical and chemical characteristics of the sediments and water of the sites where the organisms were collected showed that together, PCO1 and PCO2 explained 61% of the total variation obtained among the different areas (Figure 7A). The obtained results revealed that the samples were separated into three main groups. PCO1 explained 42% of total variation separating the samples collected in autumn (except site SJ), the samples from sites SJ and CN in winter, and the samples from sites CD and CN in spring and summer. The major elements Al, Fe, K, Mg, Na, and P and the physical and chemical parameters DO, % of fine particles, and % OM presented a positive correlation with axis 1 (r > 0.7), indicating that higher percentages of these characteristics are responsible for the higher concentrations of these major elements in the sediments (Figure 7A). Axis 2 explains 19% of the total variation separating sites M and SJ in autumn and site T in spring and summer, and these variables are on the positive side of the graph. The major elements Al, Ca, Fe, K, and Na present in the sediments had a positive correlation with this axis (r > 0.7). Site CD in winter and site CN in spring and summer were separated on the negative side of axis 2, and the major elements Mg and P and the % of fine particles, % OM, DO, and pH were negatively correlated with this axis (r > 0.7) (Figure 7A).

Regarding the PCO of the physical and chemical characteristics and trace elements of the sediments (Figure 7B), axis 1 showed 56.7% of the total data variation, separating the sites in summer and spring (except site CD), site SJ in autumn, and sites CD and T in winter on the positive side from the remaining sites on the negative side. The trace elements As, Ba, Be, Co, Cr, Cu, Li, Mn, Ni, Sn, Tl, V, and Zn and the % of fine particles and % of OM were negatively correlated with axis 1 (Figure 7B). The PCO for axis 2 showed 10.9% of the total data variation, with the sites in winter, site T in autumn, sites CD and CN in spring, and site CN in summer appearing on the positive side and separated from the remaining sites on the negative side. DO, the % of fine particles, % OM, and trace elements such as Cu, Cr, Mn, Ni, Pb, V, and Zn were strongly correlated with this side of axis 2. The trace elements As, Ba, Be, Co, Li, Sn, Tl, and W and temperature were negatively correlated with axis 2 (Figure 7B).

#### 3.7.2. Biochemical Parameters and Physical–Chemical Data

**Figure 8:**
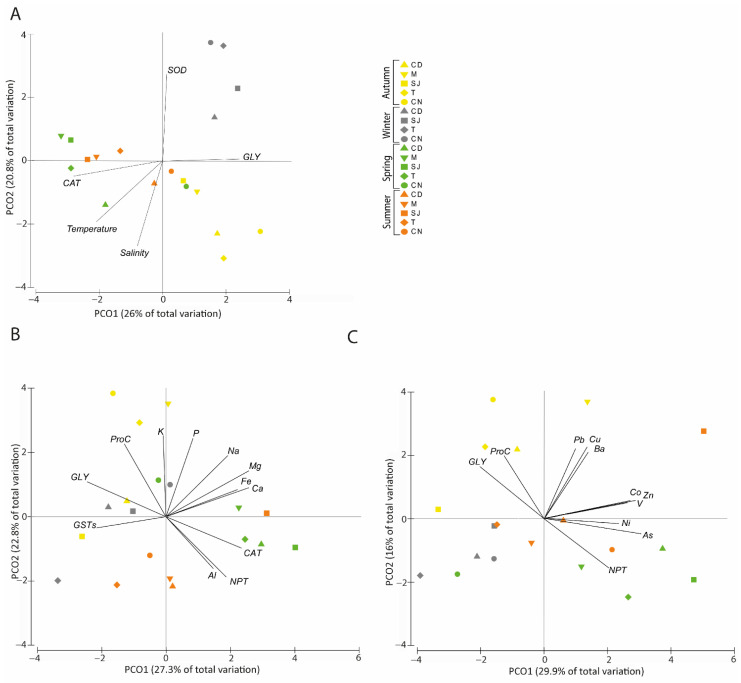
Principal coordinates ordination analysis (PCO) of (**A**) biochemical and physical–chemical parameters, (**B**) soluble fraction of *Diopatra neapolitana* tissues and biochemical parameters, and (**C**) soluble fraction of *Diopatra neapolitana* tissues and biochemical parameters. (r > 0.7). CD, M, SJ, T, and CN represent the sites Cale do Ouro, Murtosa, São Jacinto, Torreira, and Costa Nova, respectively. A, W, Sp, and Su represent the seasons of the year: autumn, winter, spring, and summer.

Principal coordinates ordination (PCO) demonstrated that together, PCO1 and PCO2 explained 46.8% of the total variation obtained among the environmental data and biochemical parameters among the different areas (Figure 8A). PCO1 described 26% of the total data variation separating autumn and winter (positive side) from spring and summer (negative side), with the exception of site CN, which was entirely on the positive side. The obtained results revealed that the sampling sites were grouped together according to season, with samples taken from different areas during the same season clustering together, with the exception of site CN. The GLY content presented a higher correlation (r > 0.7) with the positive side of PCO1. On the other hand, high levels of CAT activity were strongly correlated with the negative side of PCO1 and are presented as high temperature and salinity values (Figure 8A), with the polychaetes collected in spring and summer presenting higher values of CAT activity. PCO2 showed 20.8% of the total variation, separating the sites in winter, sites M and SJ in spring and summer, and site T in summer on the positive side and presenting a positive correlation with SOD activity. On the other hand, higher CAT activity and higher temperature and salinity levels were correlated with the negative side of PCO2 (Figure 8A). 

#### 3.7.3. Elements and Biochemical Parameters Correlation

##### Major Elements

PCO analysis of the major elements accumulated in the soluble fraction and biochemical parameters demonstrated that PCO1 showed 27.3% of the total variation, with the sites in spring (except site CN); sites CD, M, and SJ in summer; site M in autumn; and site CN in winter being on the positive side. NPT and CAT activity and most of the major elements were strongly correlated with this side as well (Figure 8B). The GLY, GST, and ProC levels and the major element potassium (K) were found to be correlated with the negative side of PCO1. PCO2 showed 22.8% of the total variation, with autumn (except SJ) and winter (except T) on the positive side and separated from spring (except sites CN and M) and summer (except site SJ), which were on the negative side. GLY, ProC, and most of the major elements were correlated with the positive side, while CAT, GSTs, NPT, and Al were correlated with the negative side (Figure 8B).

##### Trace Elements

Regarding trace element accumulation on the soluble fraction and biochemical parameters, PCO analysis demonstrated that PCO1 explained 29.9% of total data variation, with the summer (except M and T) and spring (except CN) samples on the positive side being related to higher trace element (As, Ba, Co, Cu, Ni, Pb, V, and Zn) concentrations and NPT activity (Figure 8C). The winter and autumn samples on the negative side were mainly related to the biochemical parameters GLY and ProC. PCO2 explained 16% of the variation, with the samples from winter, spring, and summer (except site SJ) on the negative side being correlated with the As and Ni accumulation levels in the soluble fraction. Samples collected in autumn and from site SJ in summer were on the positive side of this axis and were correlated with Ba, Co, Cu, Pb, V, and Zn and GLY and ProC (Figure 8C).

## 4. Discussion

The ability of polychaetes to bioaccumulate substantial amounts of elements from water and sediments has been previously demonstrated in other studies (e.g., [13,45,65,66]). However, information about how element bioaccumulation varies over seasons in polychaete tissues is scarce, and this study also included a wider variety of elements. Previous studies demonstrated that a high accumulation of metals and metalloids might cause deleterious effects in organisms, such as oxidative stress, which result from the overproduction and accumulation of reactive oxygen species (e.g., [67,68]). Additionally, other studies revealed that changes in abiotic conditions, such as salinity and temperature, can also impact polychaete performance [36,38]. Thus, it is expected that in environmental studies, organism performance can be influenced by contaminants but also by natural variations. Therefore, it is important to understand how the abiotic factors associated with seasonality, such as temperature and salinity, impact the response of *D. neapolitana* to contaminants.

### 4.1. Physical and Chemical Characteristics of Sediment Samples

Regarding the major and trace element concentrations in sediments, in this study, it was demonstrated that in general, sediments with a higher % OM and % of fine particles presented higher concentrations of major and trace elements. These results lead to the conclusion that, in this study, the element concentration in sediments is linked to higher levels of % OM and % fine particles. Element availability can depend on the pH, % OM, salinity, Eh, and sediment grain size as well as the interaction between these parameters. This might interfere with the solid and solution phases of the elements [69,70,71]. Although element availability might be related to the parameters mentioned above, it might also be related to seasonal and spatial variation. In this study, differences were observed in the element concentration among areas and seasons, showing that in general, autumn was the season in which higher element concentrations were observed, and CD was the site where elements were observed in higher concentrations. These results allow us to conclude that element concentrations are associated with changes in the sediment characteristics among locations. Previous works also demonstrated that the physical–chemical characteristics of sediment influenced the element concentration in that sediment [13,72,73,74,75]. Some of these studies were conducted at the Ria de Aveiro lagoon [13,73]. However, element availability might also change due to anthropogenic activity, which can alter major and trace elements concentrations among areas [76]. Overall, all of the tested elements were found in higher concentrations during autumn in CD, as previously mentioned. This might have happened due to the high % of OM and % of fine particles that occurred in that season.

### 4.2. Polychaete Bioaccumulation

The elements that bioaccumulated more in the insoluble and soluble fractions were not the same elements that occurred in higher concentrations in the sediments. Regarding the major elements, site M during autumn and sites CD and T during winter had higher levels of major elements compared to the remaining sites; however, concerning trace elements, site CN during summer had higher levels of element bioaccumulation (Table S3). These results allow us to conclude that element bioaccumulation in tissue samples from organisms was not linked to the total concentration in sediments but to their availability, as previously stated by other authors [13,72]. Other authors have also observed that element bioaccumulation in the tissues from the polychaetes *D. neapolitana* and *Hediste diversicolor* were not correlated with the element concentration in sediments [13,65,77,78]. The results obtained in this study showed a clear separation between element bioaccumulation in the insoluble and soluble fractions, and those elements were not absorbed in the same amounts (Tables S4 and S5). Wallace and Luoma showed that element distribution in organisms occurs in the insoluble and soluble fractions [19]. These authors also stated that the insoluble fraction retains more elements in the rich granules and in cellular debris, while elements in the soluble fraction are found in the cytosol as free ions or bound to cytoplasmatic molecules such as metallothioneins and metallothionein-like proteins and heat-sensitive proteins [50]. However, besides the presence of these metallothioneins, most of the elements present in the soluble fraction are readily available for trophic transfer. Therefore, it is essential to analyse element bioaccumulation in both fractions separately to understand the interaction between species [66].

### 4.3. Biochemical Responses

#### 4.3.1. Metabolism-Related Parameters

Concerning the performance of the organisms, proteins play an important role in biological processes, and they assist in various chemical processes that occur in living organisms, such as repair and maintenance, energy, transport, and molecule storage [52]. The availability of food can be associated with alterations in protein content; therefore, it is expected that during spring, the protein content might be higher (Table S6). In fact, Helland et al. (2003) [79] showed that the protein content in copepod *Calanus finmarchicus* females decreased as the organisms starved and was higher in organisms during spring than in autumn, when they had more food available. Overall, in this study, protein content was mainly higher during spring than in autumn, similar to what happened in [79], leading us to conclude that these differences might mainly occur due to food availability.

GLY is a carbohydrate that plays an important role as an energy source and in energy storage in organisms. Previous studies demonstrated that marine organisms may limit the expenditure of energy reserves by reducing their metabolic capacity under stressful conditions [53] as exposure to metals and temperature increases [45,80]. In our study, the GLY content presented the lowest values in spring when the temperature started to increase (Table S6). These observations may indicate that in this season, these organisms may use more energy to activate metabolic pathways, such as detoxification pathways for antioxidant defence [45,80]. These low values might also be related to their reproduction period since organisms start to develop gametes in this season [32,81]. During autumn, polychaetes bioaccumulate elements such as Cu, Mn, Pb, and Sn. Higher levels of GLY content were also detected, indicating that polychaetes preserve their energy reserves when exposed to these elements.

ETS measures the potential metabolic activity in organisms in response to environmental changes (e.g., [82,83,84]). Previous studies have reported that in the amphipod *Gammarus fossarum* [83] and in the mussel *Mytilus galloprovincialis* [85], ETS activity increased as the temperature increased. When exposed to arsenic [86], the clam *Ruditapes philippinarum* also increases the ETS levels. In this study, summer had high temperatures as well as the highest bioaccumulation of trace elements such as As, Mn, P, and Zn in the insoluble and soluble fractions, and the high levels of ETS activity observed in this season might be associated with detoxification pathways, as per the increase in SOD and GST activity (Table S6). During winter, ETS activity was also high, which may be related to an increase in metabolic activity due to unfavourable conditions such as low salinity and temperatures and due to high concentrations of elements as As, Cu, Cr, Ni, Pb, and Zn. Autumn was the season with the lowest metabolic activity. Sokolova (2013) [87] stated that to fight against oxidative stress situations, organisms (including polychaetes) may reduce their metabolism to preserve their energy reserves. The PROT and GLY content were higher during this season, suggesting that *D. neapolitana organisms* reduce their metabolism to preserve their energy reserves.

#### 4.3.2. Antioxidant Defences

Antioxidant systems are efficient protective mechanisms against ROS. CAT is an antioxidant enzyme that is present in the peroxisome of cells and is responsible for the degradation of hydrogen peroxide (H_2_O_2_), converting it into water (H_2_O) and oxygen (O_2_) [88]. Pires et al. (2017) [13] observed an increase in CAT activity in *D. neapolitana* as well as higher As bioaccumulation. Andrade et al. (2019) [89] obtained similar results with the mussel *Mytilus galloprovincialis* when it was exposed to warming waters. In general, our results show that enzyme activity was higher in spring and summer (Table S6). Generally, in these seasons, the temperature and salinity were higher, and a high correlation was observed between higher CAT activity and salinity and increase in the water temperature (Figure 5A). Moreover, during these seasons, there was high As and Zn bioaccumulation at all sites, which might be also associated with the increase in CAT activity [13] (Figure S3 and Figure 5).

SOD is an enzyme that catalyses the dismutation of reactive oxygen species (ROS), superoxide radicals, in H_2_O_2_ and O_2_, providing an important defence against oxidative damage [57]. Bhuiyan et al. (2021) [20] observed that low temperatures increased the activity of this enzyme in *Hediste diversicolor*. Freitas et al. (2015) [39] demonstrated that SOD activity increased in the polychaete *D. neapolitana* when exposed to low salinity levels. In this study, SOD activity increased during winter when the salinity values were low (Table S6). Gomes et al. (2013) [90] and Bocchetti et al. (2017) [91] also reported high SOD activity in the polychaetes *Hediste diversicolor* and *Sabella spallanzanii* when exposed to low temperatures and element bioaccumulation. In addition, the bioaccumulation of the trace elements Li, V, Cr, Ca, Tl, and K was higher in winter. Overall, the high values of SOD activity might be associated with low temperatures and salinity and the bioaccumulation of trace elements.

GST enzymes are a group of isoenzymes involved in the detoxification of the xenobiotics and metabolites produced from oxidative stress as lipid peroxide by-products and are widely used to assess the detoxification capacity of organisms [58].Higher activity of this enzyme prevents cellular damage, justifying the low levels of LPO observed in this study. Magalhães et al. (2019) [21] observed high GST activity in the polychaete *Nephtys cirrosa* when under high salinity levels. In this study, GST activity was higher during summer, which was when the highest salinity levels were reported, suggesting that GST activity also might be associated with this abiotic factor (Table S6). 

NPTs are a non-enzymatic defence and participate in many cellular reactions in which they search for ROS to eliminate them [92]. In this study, NPT levels were mostly higher during spring and summer, similar to CAT activity (Table S6). In these seasons, high temperatures and As and Zn bioaccumulation were observed, leading to the conclusion that these factors cause higher levels of ROS production, justifying the high levels of these parameters.

#### 4.3.3. Oxidative Damage Endpoints

In relation to membrane damage, LPO is the oxidation of lipids through ROS, which have targets directly on a membrane’s polyunsaturated fatty acids [62]. A study conducted by Pires et al. (2017) [13] with the polychaete *D. neapolitana* observed an increase in LPO levels and SOD activity in organisms with higher As and Cu accumulation. In this study, SOD activity was high during winter; however, the increase in its activity was not enough to prevent lipid damage (Table S6). During winter, the lowest temperature and salinity values were also observed, which might also contribute to ROS formation and thus to membrane damage. The lowest LPO value was observed during the summer at the SJ site, and this value might be related to the high activity of the CAT and NPT levels, which were able to prevent damage caused by ROS. 

Carbonylated proteins (ProC) are formed when the oxidation of amino acid residues occurs, and through these carbonyl groups, it is possible to determine if protein oxidation has occurred [63,64]. When exposed to cadmium, the clam *Ruditapes decussatus* presents higher ProC levels in its digestive gland [44]. However, Morosetti et al. (2020) [85] did not observe significant changes in ProC in the mussel *M. galloprovincialis* when it was exposed to cerium (Ce) oxide nanoparticles and mercury (Hg). Moreover, the same authors also observed a decrease in ETS activity, indicating that low element accumulation did not lead to ROS production, and the organisms continued to use reserve energy and did not damage the proteins in the organisms. In this study, the ProC levels that were obtained did not vary significantly, except for in autumn, where the highest values were observed (Table S6). In this season, the GST levels were also high, leading us to conclude that the increase in salinity and element bioaccumulation might have contributed to the production of ROS, which induced protein damage. This phenomenon could lead to irreversible damage and possible cellular death [93,94].

### 4.4. Elements and Biochemical Parameters Correlation

According to the PCO related to the soluble fraction of major elements, NPT and CAT activity are enhanced in spring and summer, and the major element present in those seasons is Al, which corroborates the idea that the activity of these enzymes is related to the presence of this element. Although Ca, Fe, and Mg had higher insoluble fraction levels in winter, these elements had higher levels during spring and summer in the soluble fraction of the tissues. This might indicate that these elements bioaccumulate more in the cytosol than in the membrane when the temperature is higher, causing deleterious effects since bioaccumulation into the cytosol can be toxic, and this toxicity can enter the food chain, causing toxicity to other organisms [13].

Trace elements are more available than major elements, but they are available in smaller quantities. However, these smaller quantities can be more toxic than major elements [95]. In general, the organisms bioaccumulated more As and Zn in spring and summer, both in the soluble and insoluble fractions, and similar to major elements, this was correlated with higher CAT and NPT values in these seasons. These can be very toxic to organisms when they enter the cytosol, and so, the organisms must activate antioxidant defences to eliminate the ROS. In autumn, both fractions had higher bioaccumulation of Cu, Mn, Pb, and Sn and showed higher levels of GLY and ProC. 

In general, bioaccumulation by elements can trigger a reaction that allows organisms to detoxify these components so that they can survive in their environment. This study showed that between major and trace elements, there is no group that accumulates more than the other since both types of elements accumulate, and generally, the most significant bioaccumulation in tissues occurs in the same seasons. The soluble and insoluble fractions do not bioaccumulate elements in the same way; however, they contain cell detoxification pathways. 

## 5. Conclusions

Major and trace elements are naturally present in the environment; however, they can cause toxicity to organisms at higher levels. The objective of this study was to characterize in terms of the element concentrations of the sediments from five different sites along the Ria de Aveiro lagoon during the four seasons of the year and to determine element bioaccumulation in *Diopatra neapolitana* tissues, how it varies among seasons, and what impacts element bioaccumulation might have on organisms. Higher element concentrations in the sediments occurred when there was a higher percentage of fine particles (% of fine particles) and organic matter (% OM). Overall, the results obtained in this work demonstrated that autumn was the season when elements concentrated in the sediments the most in, especially in Cale do Ouro. However, regarding tissue samples, summer was the season in which the organisms accumulated more elements, and this bioaccumulation occurred when the salinity and temperature were at their highest. Additionally, winter was the season in which higher element bioaccumulation occurred due to the low salinity and temperature levels; however, the elements that were accumulated the most during summer and winter were different. As and Zn accumulated more in summer, and Cu, Mn, Pb, and Sn accumulated more in winter. These results also indicate that these abiotic factors influence element availability, as previously reported by other authors. 

The results obtained here showed that the element bioaccumulation patterns in polychaete tissues were not related to their concentrations in sediments, reflecting other environmental parameters (higher fine particle content and organic matter in sediments and salinity and temperature changes) that influence element availability. Moreover, the elements accumulated differently among the fractions in the polychaete tissues, with the insoluble fraction bioaccumulating more elements than the soluble fraction.

Regarding antioxidant defences, in general, higher enzyme activity was observed during spring and summer due to higher temperatures and element bioaccumulation. Metabolism-related parameters had higher levels in spring and autumn, mainly due to element bioaccumulation. Oxidative damage was higher during winter, and this mainly occurred because of the physical and chemical characteristics of the sampling sites, as the salinity and temperature decreased during that time. Additionally, temperature and salinity influenced the performance of the organisms.

Studies involving element accumulation during different seasons are important because they show how these organisms respond to the changes that occur during the year, as these abiotic factors influence the geochemistry of elements as well organism performance.

Overall, abiotic factors significantly affect organisms in systems with low contamination, such as Ria de Aveiro, which was demonstrated in this study, and we were able to notice that the element concentrations in the sediment samples and the element bioaccumulation in the tissues also varied throughout the year. 

## Data Availability

Not applicable.

## Supplementary Materials

The following supporting information can be downloaded at https://www.mdpi.com/article/10.3390/biology11081153/s1, Figure S1: Map of the sampling site from Google Earth, Figure S2: Heatmap comparing major elements bioaccumulation: (**A**) in the insoluble fraction and (**B**) in the soluble fraction of tissue samples. CD, M, SJ, T and CN represents sites Cale do Ouro, Murtosa, São Jacinto, Torreira and Costa Nova, respectively. A, W, Sp and Su represent the seasons of the year: autumn, winter, spring and summer. I and S represents the initials for the insoluble and soluble fractions. Figure S3: Heatmap comparing trace elements bioaccumulation (As, Ba, Be, Cd, Co, Cr, Cu, Li, Mn, Ni, Pb, Sb, Sn, Tl, V, W and Zn): (**A**) in the insoluble fraction and (**B**) in the soluble fraction of tissue samples. CD, M, SJ, T and CN represents sites Cale do Ouro, Murtosa, São Jacinto, Torreira and Costa Nova, respectively. A, W, Sp and Su represent the seasons of the year: autumn, winter, spring and summer. I and S represents the initials for the insoluble and soluble fractions. Table S1: Physical and chemical characteristics of the sampling areas, Table S2: Major elements concentration (mg kg^−1^ dry weight) in sediments per season among the sampling areas. For each element concentration, significant differences (*p*≤0.05) among areas and seasons are represented with different letters (a–e). A—Autumn, W—Winter, Sp—Spring, Su—Summer. Table S3: Major elements concentration (mg kg^−1^ dry weight) in polychaetes per season among the sampling areas. For each element concentration, significant differences (*p*≤0.05) among areas and seasons are represented with different letters (a–e). A—Autumn, W—Winter, Sp—Spring, Su—Summer, sol—soluble fraction, Ins—Insoluble fraction. Table S4: Trace elements concentration (mg kg^−1^ dry weight) in sediments per season among the sampling areas. For each element concentration, significant differences (*p*≤0.05) among areas and seasons are represented with different letters (a–f). A—Autumn, W—Winter, Sp—Spring, Su—Summer, sol—soluble fraction, Ins—Insoluble fraction. Table S5: Trace elements concentration (mg kg^−1^ dry weight) in polychaetes per season among the sampling areas. For each element concentration, significant differences (*p*≤0.05) among areas and seasons are represented with different letters (a–e). A—Autumn, W—Winter, Sp—Spring, Su—Summer, sol—soluble fraction, Ins—Insoluble fraction. Table S6: Resume of the expected results, based on the literature, of the impact of seasonal and element bioaccumulation on the biochemical markers analysed in the present study and the observed results [10,13,20,40,44,45,46,79,80,83,85,89].
