# Supplementary material for: Interplay of Seasonality, Major and Trace Elements: Impacts on the Polychaete Diopatra neapolitana
Source: Biology (Basel). 2022 Jul 31;11(8):1153. doi: 10.3390/biology11081153 (PMC9404888; doi:10.3390/biology11081153)
Supplement: Supplementary file 1 [file biology-11-01153-s001.zip › biology-1671597-supplementary.pdf]

biology

MDPI

Supplementary Materials

Interplay of Seasonality, Major and Trace Elements: Impacts on the Polychaete *Diopatra neapolitana*

Valéria Giménez, Paulo Cardoso, Carina Sá, Carla Patinha, Eduardo Ferreira da Silva, Etelvina Figueira and Adília Pires

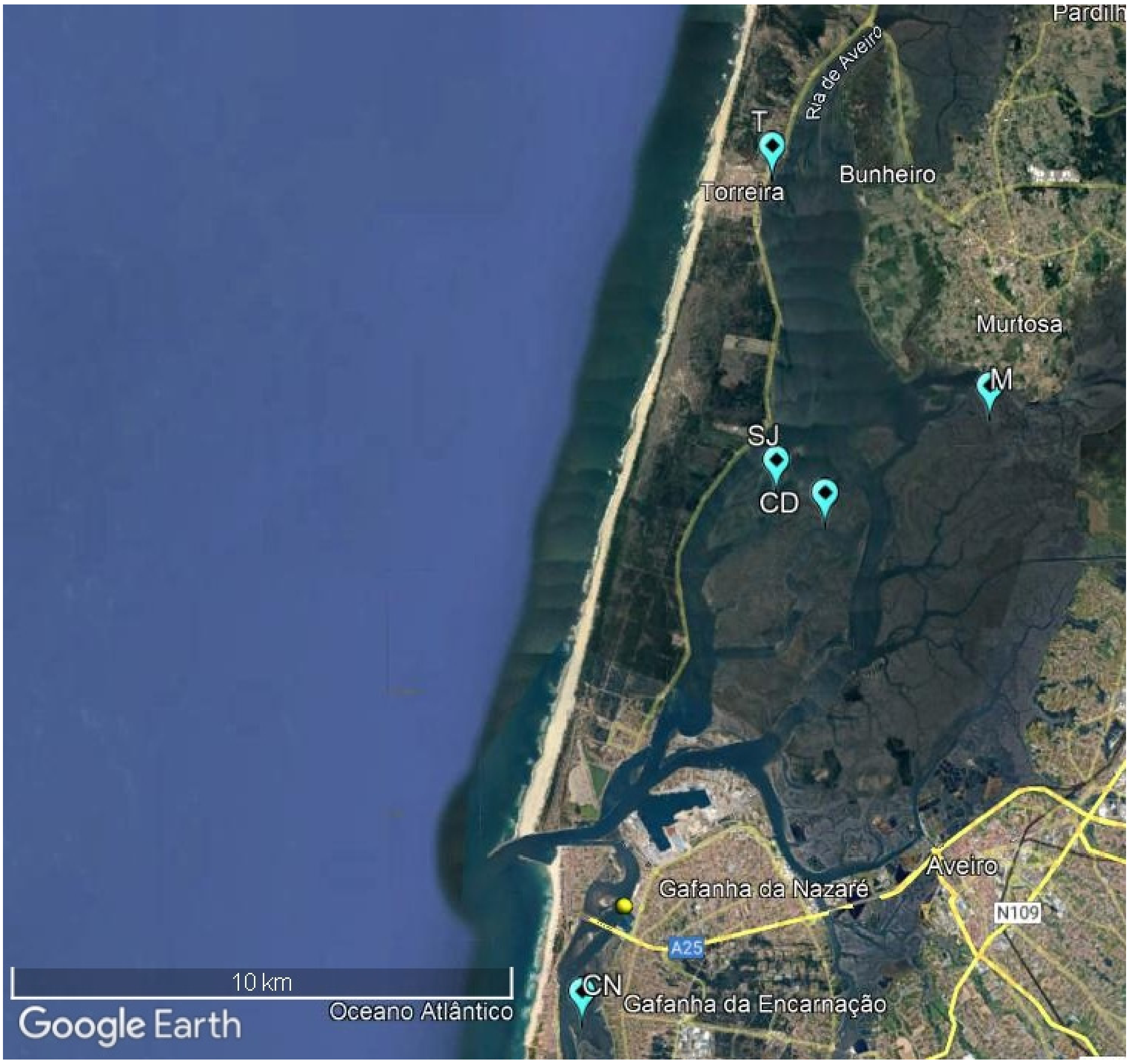

Figure S1. - Map of the sampling site from Google Earth. CD: Cale do Ouro; M: Murtosa; SJ: São Jacinto; T: Torreira; CN: Costa Nova.

A

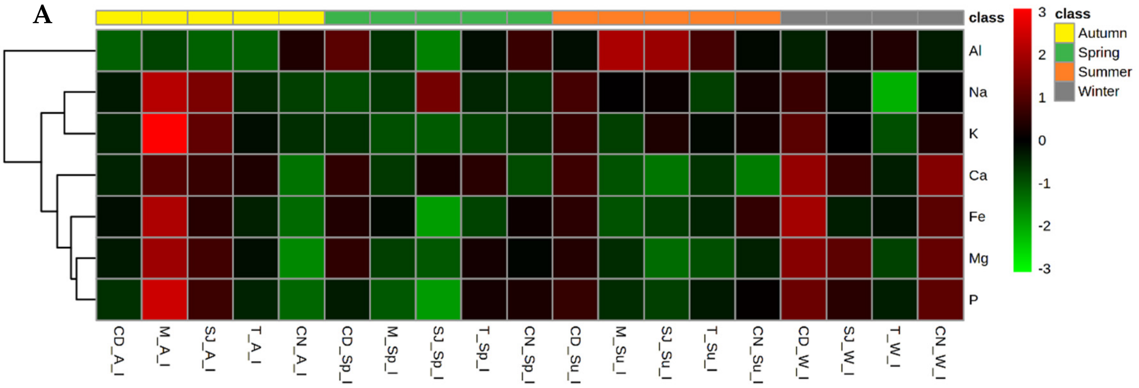

B

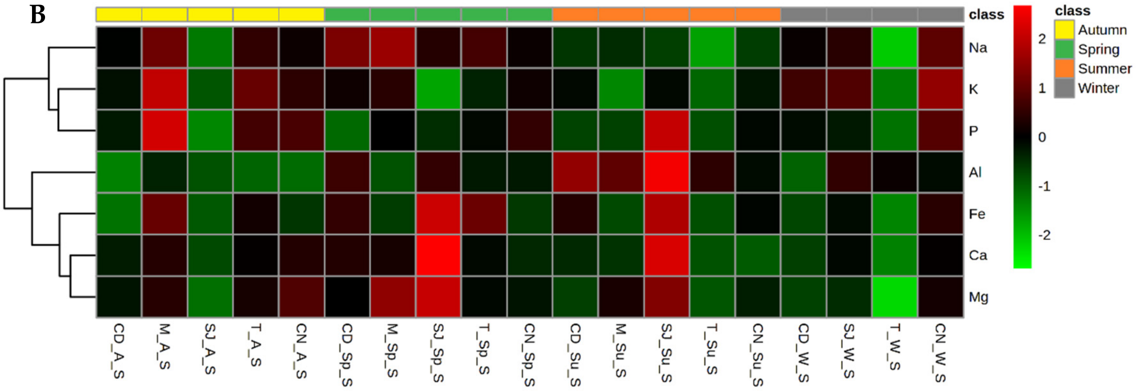

Figure S2. – Heatmap comparing major elements bioaccumulation: A- in the insoluble fraction and B- in the soluble fraction of tissue samples. CD, M, SJ, T and CN represents sites Cale do Ouro, Murtosa, São Jacinto, Torreira and Costa Nova, respectively. A, W, Sp and Su represent the seasons of the year: autumn, winter, spring and summer. I and S represents the initials for the insoluble and soluble fractions.

A

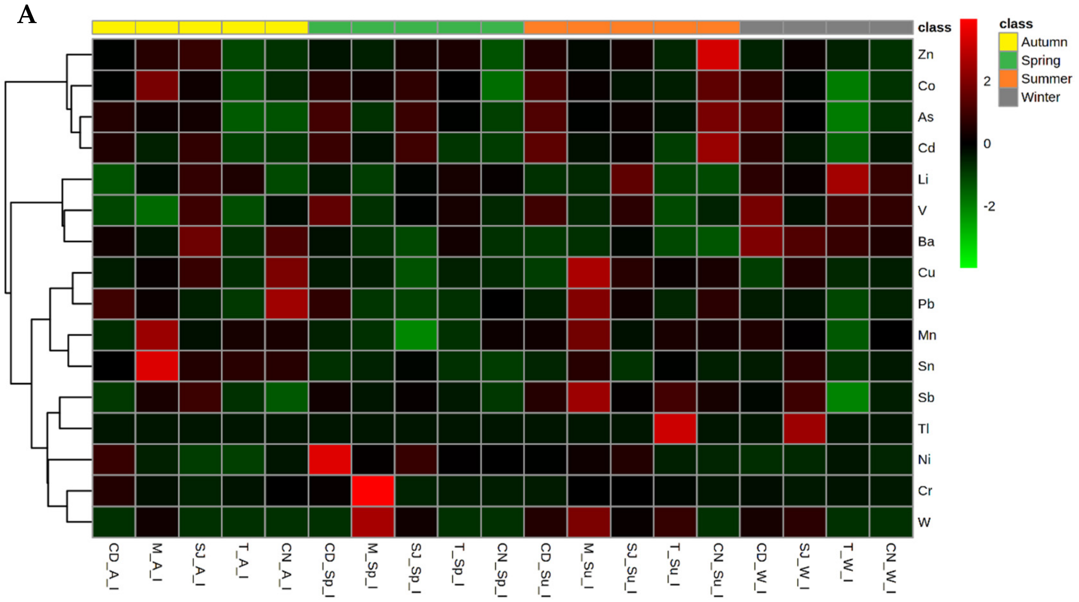

B

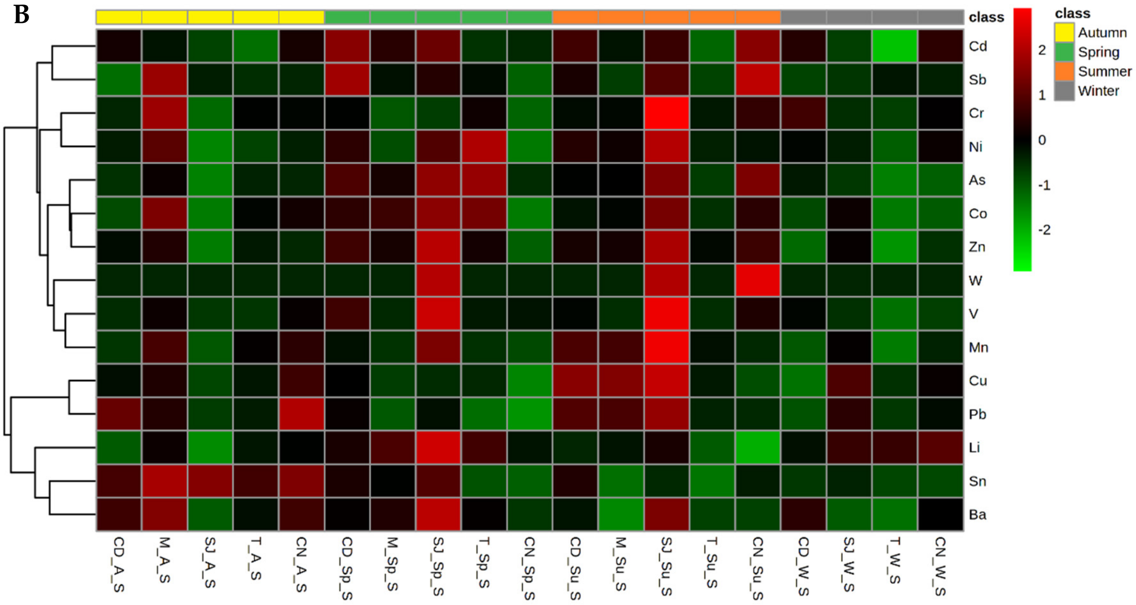

Figure S3. – Heatmap comparing trace elements bioaccumulation (As, Ba, Be, Cd, Co, Cr, Cu, Li, Mn, Ni, Pb, Sb, Sn, Tl, V, W and Zn): A- in the insoluble fraction and B- in the soluble fraction of tissue samples. CD, M, SJ, T and CN represents sites Cale do Ouro, Murtosa, São Jacinto, Torreira and Costa Nova, respectively. A, W, Sp and Su represent the seasons of the year: autumn, winter, spring and summer. I and S represents the initials for the insoluble and soluble fractions.

Table S1. Physical and chemical characteristics of the sampling areas.

Area	Season	Salinity	Dissolved oxygen (mg/L)	Redox potential (mV)	pH	Temperature (°C)	% Organic matter	% Fines
Cale do Ouro (40.7007920°, -008.6859120°)	autumn	36.39	6.72	86.23	8	16.4	6.30	53.20
	winter	34.25	8.91	135.27	7.99	13.4	1.87	12.33
	spring	37.43	7.11	83.87	7.93	18.37	1.90	7.09
	summer	38.97	8.73	88.37	8.15	22.7	3.03	17.94
Murtosa (40.7198333° -008.6470167°)	autumn	38.02	7.15	106.43	7.92	18.67	2.41	35.16
	winter	-	-	-	-	-	-	-
	spring	36.04	5.204	98.98	7.65	20.37	3.61	9.15
	summer	38.73	6.63	147.57	7.93	21.9	2.28	10.48
São Jacinto (40.7066850°, -008.6973950°)	autumn	33.87	8.56	143.67	7.99	17.43	1.64	44.77
	winter	29.21	6.7	110.37	7.89	12.23	2.79	33.85
	spring	35.26	6.03	205.07	7.81	20.9	0.68	5.50
	summer	37.58	7.16	67.27	8.27	23.7	0.77	4.41
Torreira (40.7627500°, -008.6982667°)	autumn	34.58	8.73	139.23	7.8	16.53	1.51	20.46
	winter	23.78	5.91	101.87	7.77	10.97	2.44	29.31
	spring	36.92	5.95	255.57	7.8	19.37	2.07	27.82
	summer	37.74	7.1	130.27	8	22.3	1.84	5.89
Costa Nova (40.6118056°,	autumn	34.68	6.9	189.8	7.92	16.53	1.34	4.35
	winter	19.79	7.94	186.5	8.12	13.13	1.26	13.04
	spring	36.29	9.75	113.26	8.22	20.17	1.52	24.24

- 008.7433056°)	summer	38.97	6.72	86.23	8	22.83	6.30	53.20

Table S2. Major elements concentration (mg kg^{-1} dry weight) in sediments per season among the sampling areas. For each element concentration, significant differences ($p \leq 0.05$) among areas and seasons are represented with different letters (a-e). A – Autumn, W – Winter, Sp – Spring, Su – Summer.

Sample	Al	K	Ca	Fe	Na	Mg	P
CD_A	11973.39[a]	3818.84[a]	4626.38[a]	20545.94[a]	7094.46[a]	6009.99[a]	366.73[a]
CD _W	3586.86[b,c,e]	1248.76[b,c]	5736.36[a]	6169.57[b,e]	2072.11[b,d]	1754.97[b,c]	108.27[b]
CD _Sp	4996.84[b]	1648.61[b,c]	2815.09[b]	8289.60[b]	4109.62[c]	2527.62[b,d]	200.09[c]
CD _Su	4461.45[b]	1526.58[b,c]	1968.92[b]	7363.16[b]	3209.38[b,c]	2242.91[b]	177.91[b,c]
M_A	5183.25[b]	1835.05[b]	9551.52[c]	8181.03[b]	3850.49[c]	2717.66[b,d]	250.15[a,c]
M_Sp	2363.78[c,e]	999.334[c,d]	17499.72[d]	3863.59[c]	2417.46[b]	1440.36[c]	160.30[b,c]
M_Su	4001.42[b,c]	1517.467[b,c]	4442.08[a]	6715.70[b,e]	3323.69[b,c]	2188.91 [b,c]	113.37[b]
SJ_A	5188.14[b,d]	1842.62[b,c]	13506.44[c,d]	8377.07[b]	3521.34[b,c]	2785.15[b,d]	222.67[a,c]
SJ _W	7990.52[d]	2661.83[a,b]	9821.45[c]	12564.44[d]	4130.32[c]	3643.21[d]	279.47[a,c]
SJ _Sp	4417.26[b]	1761.83[b,c]	12841.27[c,d]	7003.31[b,e]	2845.13[b,c]	2265.87[b]	175.83[b,c]
SJ _Su	3340.13[c]	1399.05[b,c]	21367.20[e]	5303.72[e]	2901.11[b,c]	1830.49[b,c]	188.23[b,c]
T_A	7185.59[d]	2199.54[b]	16126.17[d]	11231.76[d]	4616.23[c]	3379.08[d]	281.75[a]
T _W	1724.88[e]	577.73[d]	2637.61[b]	2883.77[c]	1364.14[d]	947.02[c]	57.08[d]
T _Sp	4479.53[b]	1406.0[c]	8074.06[c,e]	6969.12[b,e]	2995.63[c]	2147.70[b,c]	154.66 [b,c]
T _Su	3599.85[b]	1195.15[c]	4462.39[a]	5833.10[e]	2580.21[b,c]	1777.42[b,c]	148.24 [b,c]
CN_A	6241.8[d]	2423.66[a,b]	7547.61[e]	11116.12[d]	3578.53[b,c]	3419.09[d]	201.61[c]
CN_W	7881.56[d]	2604.91[a,b]	7931.24[e]	13047.55[d]	3506.99[b,c]	3900.08[d]	199.22 [c]
CN_Sp	4116.70[b]	1536.36[b]	4031.67[a]	6932.87[b,e]	2933.87[b]	2216.99[b]	112.63[b]
CN_Su	3833.76[b,c]	1441.20[b]	6063.44[a,e]	6623.15[b,e]	2930.53[b]	2124.93[b]	171.49[b,c]

Table S3. Major elements concentration (mg kg^{-1} dry weight) in polychaetes per season among the sampling areas. For each element concentration, significant differences ($p \leq 0.05$) among areas and seasons are represented with different letters (a-e). A – Autumn, W – Winter, Sp – Spring, Su – Summer, sol – soluble fraction, Ins – Insoluble fraction.

Sample	Na Sol	Na Ins	Al Sol	Al Ins	K Sol	K Ins	Ca Sol	Ca Ins	Fe Sol	Fe Ins	Mg Sol	Mg Ins	P Sol	P Ins
CD_A	2.90[a]	1.94[a]	75.20[a]	120.05[a,b]	2.59[a]	1.79[a]	0.23[a]	0.98[a]	19.15[a]	80.00[a,b]	0.24[a]	0.58[a]	596.56[a]	1295.79[a]
CD _W	2.97[a]	2.27[a]	95.49[a]	189.45[b]	3.07[a]	2.67[a]	0.19[a]	1.52[b]	23.59[a]	115.80[a]	0.21[a]	0.83[a]	614.28[a,d]	2003.55[b]
CD _Sp	3.65[a]	1.76[a]	244.22[b]	376.80[c]	2.77[a]	1.70[a]	0.30[a]	1.20[a,b]	37.62[a]	88.73[a,b]	0.25[a]	0.69[a]	474.26[b]	1388.83[a]
CD _Su	2.64[a]	2.32[a]	356.6[c]	216.67[d]	2.63[a]	2.42[a]	0.21[a]	1.25[a,b]	35.81[a]	90.45[a,b]	0.21[a]	0.67[a]	528.73[a,b]	1738.09[a,b]
M_A	3.56[a]	2.82[a]	148.23[d]	149.23[a,b]	3.97[a]	4.13[b]	0.30[a]	1.32[a,b]	44.58[a]	118.37[a]	0.28[a]	0.87[a]	1015.25[c]	2518.18[b]
M_Sp	3.85[a]	1.83[a]	109.12[a,d]	168.54[b]	2.92[a]	1.52[a]	0.28[a]	0.92[a]	24.66[a]	80.58[a,b]	0.36[a]	0.52[a]	633.81[a,d]	1142.03[a]
M_Su	2.69[a]	2.06[a]	288.79[b,c]	538.82[e]	1.92[a]	1.62[a]	0.20[a]	0.85[a]	23.06[a]	67.69[b]	0.27[a]	0.55[a]	532.58[a]	1322.22[a]
SJ_A	2.28[a]	2.55[a]	108.94[a,d]	117.42[a]	2.18[a]	2.74[a]	0.18[a]	1.23[a,b]	21.58[a]	89.74[a,b]	0.18[a]	0.71[a]	433.33[b]	1769.44[a,b]
SJ _W	3.15[a]	2.01[a]	232.17[b]	264.76[c,d]	3.20[a]	2.01[a]	0.25[a]	1.24[a,b]	29.95[a]	77.06[a,b]	0.23[a]	0.76[a]	591.69[a]	1678.40[a,b]
SJ _Sp	3.13[a]	2.52[a]	235.05[b]	87.00[a]	1.77[a]	1.45[a]	0.62[a]	1.14[a,b]	59.84[a]	55.41[b]	0.41[a]	0.49[a]	563.39[a]	904.98[a]
SJ _Su	2.58[a]	2.08[a]	507.02[e]	502.77[e]	2.62[a]	2.22[a]	0.56[a]	0.78[a]	55.40[a]	71.49[b]	0.35[a]	0.46[a]	986.86[c]	1237.62[a]
T_A	3.20[a]	1.89[a]	93.58[a]	119.63[a]	3.34[a]	1.94[a]	0.26[a]	1.16[a]	33.56[a]	76.22[a,b]	0.27[a]	0.59[a]	745.46[d]	1355.85[a]
T _W	1.90[a]	1.41[a]	191.43[b]	284.53[c,d]	1.97[a]	1.52[a]	0.13[a]	0.99[a]	17.53[a]	79.43[a,b]	0.13[a]	0.52[a]	460.17[b]	1377.02[a]
T _Sp	3.32[a]	1.90[a]	159.06[d]	216.75[d]	2.46[a]	1.61[a]	0.25[a]	1.19[a,b]	45.15[a]	70.26[b]	0.25[a]	0.64[a]	616.19[a,d]	1593.27[a]
T _Su	2.09[a]	1.80[a]	227.68[b]	341.97[c]	2.09[a]	1.95[a]	0.17[a]	0.94[a]	22.57[a]	75.77[a,b]	0.20[a]	0.50[a]	512.82[a]	1404.26[a]
CN_A	2.99[a]	1.81[a]	89.96[a]	280.55[c,d]	2.95[a]	1.72[a]	0.30[a]	0.78[a]	25.30[a]	63.74[b]	0.31[a]	0.42[a]	753.87[d]	1084.18[a]
CN_W	3.46[a]	2.06[a]	169.62[d]	197.80[d]	3.64[a]	2.24[a]	0.26[a]	1.46[b]	36.26[a]	100.17[a,b]	0.27[a]	0.77[a]	776.79[d]	1929.91[a]
CN_Sp	2.99[a]	1.86[a]	157.68[d]	320.33[c]	2.77[a]	1.73[a]	0.22[a]	0.88[a]	24.90[a]	84.69[a,b]	0.24[a]	0.60[a]	716.33[d]	1619.63[a,b]
CN_Su	2.60[a]	2.13[a]	170.18[b,d]	222.40[d]	2.55[a]	2.14[a]	0.17[a]	0.76[a]	30.66[a]	92.04[a,b]	0.24[a]	0.56[a]	621.13[a,d]	1524.77[a,b]

Table S4. Trace elements concentration (mg kg^{-1} dry weight) in sediments per season among the sampling areas. For each element concentration, significant differences ($p \leq 0.05$) among areas and seasons are represented with different letters (a-f). A – Autumn, W – Winter, Sp – Spring, Su – Summer, sol – soluble fraction, Ins – Insoluble fraction.

Sample	Cr	Ni	Cu	Zn	As	Cd	Pb	Li	Be
CD_A	19.55^{a}	14.3^{a}	16.3^{a}	122.36^{a}	14.00^{a}	0.35^{a}	22.93^{a}	51.37^{a}	1.69^{a}
CD _W	5.78^{b,c}	4.09^{b}	4.32^{b}	35.01^{b,d}	4.12^{b,c}	0.05^{b}	6.38^{b}	15.3^{b,e}	0.71^{b}
CD _Sp	2.54^{c}	1.63^{c}	1.87^{c}	14.31^{c}	2.31^{c}	0.17^{c}	3.58^{c}	6.02^{c}	0.64^{b}
CD _Su	7.81^{b,d}	5.43^{b}	6.87^{c}	54.26^{b,e}	5.37^{b,d}	0.19^{c}	10.55^{d}	18.0^{b,d}	0.56^{b}
M_A	9.46^{d}	5.54^{b}	6.99^{c}	53.98^{b,e}	5.43^{b,d}	0.13^{c}	9.62^{d}	23.35^{d}	0.76^{b}
M_Sp	3.65^{c}	2.93^{b,c}	2.49^{b,d}	29.90^{d}	3.44^{c}	0.10^{b,c}	4.70^{b,c}	11.32^{e}	0.39^{c}
M_Su	3.14^{c}	2.49^{b,c}	2.29^{d}	30.84^{b,d}	3.60^{c}	0.28^{a}	4.00^{c}	17.59^{b}	0.51^{b,c}
SJ_A	7.42^{b,d}	5.72^{b}	5.11^{b}	46.01^{b}	4.77^{b,c}	0.00^{d}	7.49^{b,d}	23.99^{d}	0.83^{b}
SJ _W	5.93^{b}	4.27^{b}	3.24^{b}	46.58^{b}	4.81^{b}	0.31^{a}	6.38^{b}	25.31^{d,f}	0.65^{b}
SJ _Sp	6.06^{b}	4.49^{b}	3.22^{b}	48.27^{b}	3.92^{c}	0.00^{d}	4.89^{b,c}	25.73^{d,f}	0.77^{b}
SJ _Su	4.69^{b,c}	3.59^{b,c}	2.47^{b,d}	33.20^{b,d}	3.58^{c}	0.08^{b}	4.37^{b,c}	20.03^{d}	0.18^{d}
T_A	10.11^{d}	8.08^{d}	9.63^{e}	67.55^{e}	7.16^{d}	0.18^{c}	11.72^{d}	27.10^{f}	1.13^{a}
T _W	2.81^{c}	2.18^{c}	2.43^{b,d}	26.01^{d}	2.02^{c}	0.00^{d}	3.29^{c}	6.99^{c}	0.00^{e}
T _Sp	6.87^{b,d}	5.07^{b}	5.48^{b,c}	56.67^{b,e}	4.43^{b,c}	0.15^{c}	6.72^{b}	17.22^{b}	0.73^{b}
T _Su	5.59^{b,c}	4.33^{b}	4.88^{d}	50.72^{b,e}	4.85^{b}	0.17^{c}	5.85^{b}	13.91^{b,e}	0.44^{c}
CN_A	10.18^{d}	7.25^{d}	6.83^{c}	51.43^{b,e}	7.60^{d}	0.09^{b,c}	8.59^{b,d}	30.48^{f}	1.16^{a}
CN_W	6.78^{b,d}	4.90^{b}	4.28^{d}	46.96^{b}	5.95^{b,d}	0.10^{b,c}	5.93^{b}	19.45^{b,d}	0.72^{b}
CN_Sp	2.76^{c}	1.85^{c}	1.70^{c}	13.54^{c}	1.96^{c}	0.05^{b}	6.68^{b}	6.76^{c}	0.35^{c}
CN_Su	7.33^{b,d}	4.94^{d}	5.59^{b,c}	33.89^{b,d}	4.75^{b}	0.08^{b,c}	7.35^{b}	16.74^{b}	0.55^{b,c}
**Sample**	**V**	**Mn**	**Co**	**Sn**	**Sb**	**Ba**	**W**	**Tl**	
CD_A	23.14^{a}	150.02^{a}	5.35^{a}	1.16^{a}	0.04^{a}	22.97^{a}	0.13^{a}	0.35^{a}	
CD _W	7.32^{b}	50.30^{b}	1.60^{b,c}	0.29^{b,d}	0.03^{a}	8.20^{b}	0.00^{b}	0.11^{b}	
CD _Sp	3.33^{c}	66.54^{c,e}	2.13^{b}	0.36^{b}	0.08^{a}	10.60^{b,d}	0.05^{c}	0.14^{b}	
CD _Su	8.69^{d}	52.76^{b,c}	1.92^{b}	0.49^{b,c}	0.08^{a}	11.43^{b,d}	0.20^{d}	0.16^{b}	
M_A	9.28^{d}	61.67^{c}	2.32^{b}	0.58^{c}	0.05^{a}	10.43^{b,d}	0.05^{c}	0.18^{b,c}	
M_Sp	4.90^{c}	33.29^{d}	1.31^{c}	0.30^{b}	0.07^{a}	9.89^{b}	0.03^{c}	0.10^{b}	
M_Su	7.81^{b,d}	54.60^{b}	2.13^{b}	0.53^{b,c}	0.04^{a}	11.61^{b,d}	0.11^{a,e}	0.18^{b,c}	
SJ_A	9.08^{d}	71.11^{e,f}	2.52^{b}	0.58^{b,c}	0.04^{a}	11.80^{b,d}	0.23^{d}	0.16^{b}	
SJ _W	8.51^{d}	77.15^{e,f}	2.06^{b}	0.70^{c}	0.03^{a}	17.67^{c,e}	0.00^{b}	0.18^{b,c}	
SJ _Sp	7.91^{d}	72.09^{e,f}	2.26^{b}	0.68^{c}	0.05^{a}	11.66^{b,d}	0.00^{b}	0.18^{b,c}	
SJ _Su	6.13^{b}	57.48^{b}	1.80^{b,c}	0.60^{c}	0.04^{a}	12.23^{d,e}	0.15^{a}	0.15^{b}	
T_A	12.30^{e}	74.37^{f}	3.28^{b}	0.56^{b,c}	0.04^{a}	14.90^{e}	0.00^{b}	0.18^{b}	
T _W	3.29^{c}	27.18^{d}	0.92^{c}	0.16^{d}	0.04^{a}	5.32^{f}	0.00^{b}	0.02^{d}	
T _Sp	7.71^{b,d}	58.47^{b}	2.06^{b,c}	0.44^{b}	0.03^{a}	12.06^{d,e}	0.04^{c}	0.12^{b}	
T _Su	6.56^{d}	53.69^{b}	1.76^{b,c}	0.29^{b,d}	0.03^{a}	10.92^{b,d}	0.08^{e}	0.11^{b}	
CN_A	13.02^{e}	94.05^{g}	3.22^{b}	0.77^{c}	0.07^{a}	16.97^{c,e}	0.07^{e}	0.25^{a,c}	
CN_W	8.71^{d}	61.43^{c}	2.24^{b}	0.44^{b}	0.04^{a}	14.97^{e}	0.08^{e}	0.33^{a}	
CN_Sp	3.10^{c}	19.80^{d}	1.97^{b,c}	0.25^{b}	0.05^{a}	11.16^{b,d}	0.03^{c}	0.15^{b}	
CN_Su	8.37^{b,d}	48.44^{b}	1.89^{b,c}	0.53^{b,c}	0.05^{a}	12.28^{b,d,e}	0.11^{a,e}	0.15^{b}	

Table S5. Trace elements concentration (mg kg^{-1} dry weight) in polychaetes per season among the sampling areas. For each element concentration, significant differences ($p \leq 0.05$) among areas and seasons are represented with different letters (a-e). A – Autumn, W – Winter, Sp – Spring, Su – Summer, sol – soluble fraction, Ins – Insoluble fraction.

Sample	Li Sol	Li Ins	V Sol	V Ins	Cr Sol	Cr Ins	Mn Sol	Mn Ins	Co Sol	Co Ins	Ni Sol	Ni Ins	Cu Sol	Cu Ins	Zn Sol	Zn Ins
CD_A	0.05^{a}	0.08^{a}	0.05^{a}	0.02^{a}	0.12^{a}	2.28^{a}	0.08^{a}	3.52^{a}	$0.09^{a,c}$	0.01^{a}	0.51^{a}	$0.07^{a,c}$	$1.41^{a,b}$	2.06^{a}	8.65^{a}	$27.31^{a,b}$
CD _W	0.07^{a}	$0.11^{a,b}$	$0.07^{a,b}$	$0.12^{b,c}$	0.21^{b}	0.86^{b}	0.27^{b}	7.18^{b}	0.20^{b}	$0.08^{b,c}$	1.02^{b}	0.32^{b}	$1.87^{a,c}$	$2.64^{a,b}$	$10.45^{a,c}$	31.33^{a}
CD _Sp	0.04^{a}	0.15^{b}	0.05^{a}	$0.13^{b,c}$	$0.05^{c,f}$	0.18^{c}	0.05^{a}	3.95^{a}	0.07^{a}	0.02^{a}	0.27^{c}	0.02^{a}	$1.00^{b,d}$	$3.48^{b,c}$	5.34^{b}	32.69^{a}
CD _Su	0.07^{a}	0.13^{b}	0.05^{a}	0.17^{b}	0.21^{b}	0.16^{c}	0.17^{c}	$4.60^{a,b,c}$	0.12^{c}	0.02^{a}	$0.40^{a,c,d}$	0.01^{a}	$1.35^{a,b}$	1.85^{a}	8.16^{a}	21.67^{b}
M_A	0.07^{a}	0.08^{a}	$0.07^{a,b}$	0.08^{c}	0.30^{e}	0.43^{d}	$0.22^{b,c}$	$4.62^{a,b}$	0.13^{c}	0.03^{a}	0.50^{a}	$0.12^{c,e}$	2.24^{c}	4.91^{c}	$7.72^{a,b}$	23.13^{b}
M_Sp	0.07^{a}	$0.14^{b,c}$	0.06^{a}	0.15^{b}	$0.17^{a,b}$	0.41^{d}	0.05^{a}	$4.75^{a,b}$	$0.09^{a,c}$	$0.05^{a,b}$	0.58^{a}	$0.07^{a,c}$	0.75^{d}	$1.61^{a,d}$	5.81^{b}	24.45^{b}
M_Su	0.09^{a}	0.12^{b}	0.05^{a}	0.03^{a}	0.1^{a}	$0.22^{c,d}$	0.32^{b}	$4.27^{a,b}$	0.13^{c}	0.02^{a}	0.51^{a}	0.01^{a}	1.10^{b}	3.06^{b}	9.36^{a}	$28.56^{a,b}$
SJ_A	0.09^{a}	0.23^{c}	0.03^{a}	0.08^{c}	0.20^{b}	0.20^{c}	0.05^{a}	2.85^{a}	0.07^{a}	0.01^{a}	0.35^{d}	$0.07^{a,c}$	1.13^{b}	1.92^{a}	4.84^{b}	24.72^{b}
SJ _W	0.10^{a}	$0.15^{b,c}$	0.05^{a}	$0.12^{b,c}$	0.27^{e}	0.35^{d}	0.07^{a}	$4.22^{a,b}$	0.08^{a}	0.02^{a}	$0.64^{a,e}$	$0.08^{a,c}$	$1.62^{a,c}$	2.05^{a}	$7.38^{a,b}$	23.29^{b}
SJ _Sp	0.08^{a}	0.11^{b}	0.10^{b}	$0.12^{b,d}$	$0.08^{a,c}$	1.13^{b}	0.05^{a}	$3.67^{a,c}$	0.15^{c}	0.06^{b}	0.78^{e}	$0.25^{b,f}$	1.54^{a}	2.15^{a}	11.82^{c}	$25.93^{a,b}$
SJ _Su	$0.09^{a,b}$	$0.09^{a,b}$	0.05^{a}	$0.13^{b,d}$	0.07^{c}	0.01^{e}	0.08^{a}	$3.45^{a,c}$	0.15^{c}	0.06^{b}	$0.38^{c,d}$	0.41^{d}	1.05^{b}	2.07^{a}	$10.04^{a,c}$	24.92^{b}
T_A	0.14^{b}	0.1^{b}	0.22^{c}	0.36^{e}	$0.16^{a,b}$	0.21^{c}	$0.10^{a,d}$	2.25^{a}	0.22^{b}	0.06^{b}	0.96^{b}	0.04^{a}	1.18^{b}	1.37^{d}	9.07^{a}	$29.81^{a,b}$
T _W	$0.09^{a,b}$	0.13^{b}	0.06^{a}	0.06^{b}	$0.17^{a,b}$	0.35^{d}	0.08^{a}	$3.43^{a,c}$	0.20^{b}	0.03^{a}	1.69^{f}	0.12^{c}	1.21^{b}	2.01^{a}	$9.96^{a,c}$	30.15^{a}
T _Sp	0.07^{a}	0.12^{b}	0.06^{a}	0.17^{b}	$0.18^{a,b}$	0.17^{c}	0.11^{d}	4.43^{c}	0.07^{a}	0.02^{a}	0.29^{c}	$0.16^{e,f}$	0.67^{d}	1.88^{a}	6.07^{b}	20.66^{b}
T _Su	0.06^{a}	$0.09^{a,b}$	0.07^{a}	0.08^{c}	0.23^{b}	0.50^{d}	0.53^{e}	4.47^{c}	0.11^{c}	0.07^{b}	0.75^{e}	$0.27^{b,f}$	1.15^{b}	$1.60^{a,d}$	$10.03^{a,c}$	30.70^{a}
CN_A	0.07^{a}	0.10^{b}	0.05^{a}	0.16^{b}	0.23^{b}	0.59^{d}	$0.40^{e,f}$	6.32^{b}	0.12^{c}	0.10^{c}	$0.66^{a,e}$	0.19^{f}	1.54^{a}	6.03^{e}	$9.95^{a,c}$	$26.90^{a,b}$
CN_W	$0.08^{a,b}$	0.18^{c}	0.27^{c}	0.07^{c}	0.01^{f}	0.36^{d}	0.36^{f}	$3.94^{a,c}$	0.20^{b}	0.10^{c}	$0.83^{b,e}$	0.13^{e}	3.03^{e}	3.20^{b}	11.41^{c}	$29.49^{a,b}$
CN_Sp	0.05^{a}	0.08^{a}	0.05^{a}	0.06^{c}	0.06^{c}	0.88^{b}	$0.11^{a,d}$	$4.64^{a,b,c}$	$0.10^{a,c}$	0.06^{b}	0.50^{a}	$0.11^{c,e}$	$1.34^{a,b}$	2.67^{b}	8.72^{a}	24.41^{b}
CN_Su	0.04^{a}	0.08^{a}	$0.08^{a,b}$	0.09^{c}	0.26^{e}	0.35^{d}	0.08^{a}	$4.55^{a,c}$	0.14^{c}	$0.05^{a,b}$	0.54^{a}	$0.08^{a,c}$	$0.95^{b,d}$	2.93^{b}	11.68^{c}	50.67^{c}

Table S5. (continued).

Sample	As Sol	As Ins	Cd Sol	Cd Ins	Sn Sol	Sn Ins	Sb Sol	Sb Ins	Ba Sol	Ba Ins	W Sol	W Ins	Tl Sol	Tl Ins	Pb Sol	Pb Ins
CD_A	$1.89^{a,b,c}$	4.60^{a}	0.04^{a}	$0.08^{a,c}$	0.03^{a}	0.02^{a}	0.01^{a}	0.01^{a}	$0.07^{a,b}$	$0.18^{a,b}$	n.d.	n.d.	n.d.	n.d.	$0.81^{a,b}$	$1.05^{a,b}$
CD _W	2.47^{b}	$4.08^{a,b}$	0.03^{a}	$0.05^{a,b}$	0.05^{a}	0.04^{a}	0.01^{a}	0.02^{a}	0.09^{b}	$0.15^{a,b}$	n.d.	0.01^{a}	n.d.	n.d.	0.49^{b}	0.74^{b}
CD _Sp	1.28^{c}	$4.20^{a,b}$	0.03^{a}	$0.09^{a,c}$	0.02^{a}	0.03^{a}	0.01^{a}	0.02^{a}	0.04^{a}	0.25^{b}	n.d.	n.d.	n.d.	n.d.	0.19^{c}	$0.55^{b,c}$
CD _Su	$1.99^{a,b}$	2.07^{c}	0.02^{a}	0.03^{b}	0.03^{a}	0.03^{a}	0.01^{a}	0.01^{a}	0.05^{a}	0.13^{a}	n.d.	n.d.	n.d.	n.d.	$0.27^{b,c}$	0.43^{c}
M_A	$1.97^{a,b}$	$2.25^{c,e}$	0.04^{a}	0.04^{b}	0.02^{a}	0.03^{a}	0.01^{a}	0.01^{a}	$0.07^{a,b}$	$0.22^{a,b}$	n.d.	n.d.	n.d.	n.d.	$0.36^{b,d}$	1.75^{d}
M_Sp	$2.08^{a,b}$	5.53^{d}	0.04^{a}	$0.09^{a,c}$	0.01^{a}	0.02^{a}	0.01^{a}	0.01^{a}	$0.06^{a,b}$	0.27^{b}	n.d.	0.01^{a}	n.d.	n.d.	0.16^{c}	$0.56^{b,c}$
M_Su	$1.82^{a,b,c}$	3.76^{b}	0.03^{a}	$0.05^{a,b}$	0.01^{a}	0.03^{a}	0.01^{a}	0.02^{a}	0.04^{a}	$0.23^{a,b}$	n.d.	0.01^{a}	n.d.	n.d.	0.53^{b}	$0.60^{b,c}$
SJ_A	1.29^{c}	1.60^{e}	0.01^{a}	0.02^{b}	0.01^{a}	0.01^{a}	0.01^{a}	0.01^{a}	0.03^{a}	$0.20^{a,b}$	n.d.	n.d.	n.d.	n.d.	0.20^{c}	0.39^{c}
SJ _W	$1.50^{a,c}$	2.83^{b}	0.04^{a}	$0.05^{a,b}$	0.01^{a}	0.02^{a}	0.01^{a}	0.01^{a}	0.05^{a}	$0.19^{a,b}$	n.d.	n.d.	n.d.	n.d.	0.32^{d}	$0.54^{b,c}$
SJ _Sp	3.35^{b}	5.40^{d}	0.07^{b}	0.10^{c}	0.02^{a}	0.01^{a}	0.02^{a}	0.02^{a}	0.05^{a}	0.15^{a}	n.d.	n.d.	n.d.	n.d.	$0.38^{b,d}$	0.95^{a}
SJ _Su	2.60^{b}	2.85^{b}	0.04^{a}	0.06^{a}	0.02^{a}	0.02^{a}	0.01^{a}	0.01^{a}	$0.06^{a,b}$	0.13^{a}	n.d.	0.03^{a}	n.d.	n.d.	$0.14^{c,e}$	0.45^{c}
T_A	4.55^{d}	5.16^{d}	0.06^{b}	0.10^{c}	0.03^{a}	0.02^{a}	0.01^{a}	0.02^{a}	$0.05^{a,b}$	0.12^{a}	0.01^{a}	0.01^{a}	n.d.	n.d.	0.30^{d}	0.40^{c}
T _W	4.75^{d}	3.74^{b}	0.03^{a}	0.04^{b}	0.01^{a}	0.01^{a}	0.01^{a}	0.01^{a}	$0.06^{a,b}$	$0.18^{a,b}$	n.d.	n.d.	n.d.	n.d.	$0.12^{c,e}$	0.46^{c}
T _Sp	$1.91^{a,b,c}$	$2.54^{c,e}$	0.03^{a}	0.04^{b}	0.01^{a}	0.01^{a}	0.01^{a}	0.01^{a}	0.04^{a}	0.13^{a}	n.d.	n.d.	n.d.	n.d.	0.08^{e}	0.69
T _Su	$2.30^{a,b}$	5.69^{d}	$0.05^{a,b}$	$0.12^{c,d}$	0.02^{a}	0.02^{a}	0.01^{a}	0.02^{a}	$0.05^{a,b}$	0.13^{a}	n.d.	0.01^{a}	n.d.	n.d.	$0.23^{c,d}$	0.54^{b}
CN_A	$2.33^{a,b}$	3.72^{b}	0.03^{a}	0.06^{a}	0.01^{a}	0.03^{a}	0.01^{a}	0.02^{a}	0.03^{a}	0.13^{a}	n.d.	0.02^{a}	n.d.	n.d.	$0.22^{c,d}$	$1.53^{a,d}$
CN_W	4.25^{d}	$4.10^{a,b}$	$0.05^{a,b}$	$0.07^{a,c}$	0.01^{a}	0.01^{a}	0.01^{a}	0.01^{a}	0.09^{b}	$0.16^{a,b}$	0.01^{a}	0.01^{a}	n.d.	n.d.	$0.73^{a,b}$	0.78
CN_Sp	$1.78^{a,c}$	$3.41^{b,c}$	0.02^{a}	0.04^{a}	0.01^{a}	0.02^{a}	0.01^{a}	0.02^{a}	0.04^{a}	0.12^{a}	n.d.	0.01^{a}	n.d.	n.d.	$0.25^{c,d}$	$0.52^{,c}$
CN_Su	4.21^{d}	6.88^{f}	0.07^{b}	0.16^{d}	0.01^{a}	0.02^{a}	0.02^{a}	0.02^{a}	0.04^{a}	0.11^{a}	0.01^{a}	n.d.	n.d.	n.d.	$0.23^{c,d}$	0.93^{a}

n.d. – not determinbelow the detection limit of the equipment).

Table S6. Resume of the expected results, based on literature, of seasonal and elements bioaccumulation impacts on biochemical markers analysed in the present study, and observed results. PROT– protein; GLY- glycogen; ETS- electron transport system; CAT- catalase; SOD- superoxide dismutase; GSTs- glutathione S-transferases; NPT- non protein thiols; LPO- lipid peroxidation; ProC- protein carbonylation.

BIOCHEMICAL MARKERS	EXPECTED RESULTS	OBSERVED RESULTS
PROT	↑ with availability of food [82].	↑ in spring and autumn due to food availability.
GLY	↑ with availability of food<br>↓ due to activation of metabolic pathways by exposure to elements and temperature and salinity increase [47], [83].	↑ in autumn and winter, which can indicate that organisms did not use this energy to activate other metabolic pathways (Sokolova, (2013)).<br>↓ lowest values during spring.
ETS	↑ due to increase of temperature [86], [88], and elements bioaccumulation [44].	↑ in summer due to increase of temperature
CAT	↑ due to high temperatures and bioaccumulation of elements [13], [92].	↑ in spring and summer where highest temperatures and salinity were observed and bioaccumulation of As and Zn.
SOD	↑ due to low temperatures [20], salinity changes [40] and elements bioaccumulation [13], [10], [44].	↑ high SOD activity in winter due to low temperatures and salinity.
GSTs	↑ activity at higher salinities levels (Magalhães et al., 2019) and bioaccumulation of elements [47].	↑ increase of GSTs activity during summer with high salinities and with high dissolved oxygen dissolved.
NPT	↑ due elements accumulation, high temperature and salinity changes	↑ higher levels during spring and summer due to high temperature and elements bioaccumulation.
LPO	↑ with elements bioaccumulation [13]), [10] and temperature and salinity alterations [40], due formation of Reactive Oxygen Species (ROS).	↑ higher levels during winter.<br>↓ lower levels during summer due to high activity of CAT and NPT.
ProC	↑ with elements bioaccumulation and temperature and salinity alterations, due formation of (ROS) [46].	↑ in autumn, with the highest values observed due to elements bioaccumulation.
